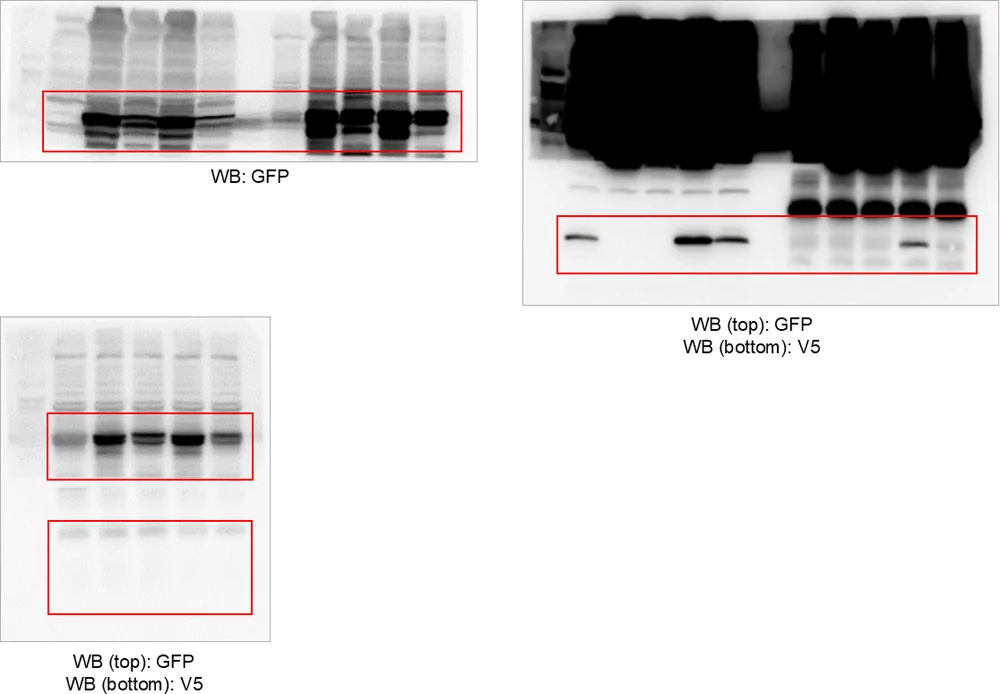# Calcium-dependent proteolysis of the chimeric androglobin reveals altered localization of its isolated globin domain

**DOI:** 10.1016/j.jbc.2026.113582

**Published:** 2026-09-18

**Authors:** Teng Wei Koay, Carina Osterhof, Angèle Clerc, David Hoogewijs

**Affiliations:** Section of Medicine, Department of Endocrinology, Metabolism and Cardiovascular System (EMC), University of Fribourg, Firbourg, Switzerland

**Keywords:** globin, oxygen-binding, calpain, calcium signaling, CAPNS1, proteolysis

## Abstract

Androglobin (ADGB), a protein essential for spermatogenesis, is one of the most structurally unusual members of the vertebrate globin superfamily. It combines a calpain-like domain with a circularly permuted globin domain containing an embedded calmodulin-binding IQ motif, an architecture suggesting complex regulatory functions that remain poorly understood. Here, we investigated whether ADGB undergoes calcium-dependent post-translational processing, as described for other calpains. ADGB displayed a characteristic proteolytic processing pattern following calcium stimulation, generating several stable cleavage products following ectopic expression in mammalian cells. *In vitro* proteolysis assays demonstrated that ADGB cleavage requires cytoplasmic factor(s) and is enhanced by Ca^2+^. Broad protease inhibitors with activity against calpains reduced ADGB processing, whereas the more selective inhibitor PD150606 and siRNA-mediated depletion of CAPN1 or CAPN2 did not substantially affect cleavage, arguing against these classical calpains as its principal mediators. In contrast, CAPNS1 downregulation reduced calcium-dependent ADGB processing in both cellular and cell-free assays, indicating a contribution of CAPNS1 without establishing a direct role in the proteolytic reaction. Domain-mapping analyses localized major cleavage regions between the N-terminal calpain-like and the globin-containing regions of ADGB. The experimentally isolated globin domain displayed enhanced interaction with calmodulin compared with full-length ADGB and preferential localization to centrosomal structures. Collectively, these findings identify calcium-dependent cytoplasmic proteolytic processing as a previously unrecognized property of ADGB and suggest that such processing may influence the biochemical accessibility and cellular behavior of its constituent domains.

Calcium is an essential divalent cation that plays a central role in intracellular signaling throughout eukaryotic biology. Intracellular Ca^2+^ functions predominantly as a second messenger coupling extracellular stimuli to diverse cellular responses including contraction, secretion, metabolism, differentiation, and cytoskeletal regulation (1). The remarkable signaling versatility of Ca^2+^ arises from its flexible coordination chemistry, allowing reversible interaction with a wide variety of calcium-binding proteins and signaling effectors. Among the major calcium-responsive protein families are the calpains, a superfamily of intracellular cysteine proteases whose activity is tightly linked to calcium signaling. Calpains are Ca^2+^-dependent, non-lysosomal neutral cysteine proteases that are broadly expressed across eukaryotes. The classical calpains CAPN1 and CAPN2 are heterodimeric complexes composed of a catalytic 80-kDa large subunit associated with a common 28-kDa regulatory small subunit, CAPNS1/CAPN4 (2). Structurally, CAPN1 and CAPN2 contain a protease core domain (CysPc domain), followed by a C2-like domain and a penta-EF-hand calcium-binding domain, whereas CAPNS1 contains a glycine-rich region and a penta-EF-hand domain involved in heterodimer stabilization (3, 4). Calcium binding induces conformational rearrangements that promote catalytic activation of the protease core (5). In addition to substrate cleavage, several calpains themselves undergo regulated proteolytic processing. CAPN1, CAPN2, and CAPNS1 all display stepwise cleavage events associated with activation or regulatory transitions (6, 7, 8). Likewise, CAPN7 undergoes proteolytic removal of its N-terminal microtubule-interacting and transport (MIT) domain (9), whereas CAPN3 displays a rapid 2-step autoproteolysis for activation, as facilitated by calmodulin binding (10, 11, 12, 13). Collectively, these observations illustrate how calcium signaling can be coupled to proteolytic regulation within the calpain superfamily and provide a rationale for investigating whether the calpain-like multidomain protein ADGB is itself subject to calcium-responsive processing.

Androglobin (ADGB) is a highly unusual and evolutionarily distinct member of the globin superfamily that was initially classified among the non-classical calpains because of its N-terminal calpain-like domain (14, 15, 16). In contrast to classical calpains, however, ADGB lacks the canonical penta-EF-hand calcium-binding domain. Instead, ADGB contains a circularly permuted globin domain harboring an embedded IQ motif predicted to interact with calmodulin. Recent biochemical and structural studies demonstrated that the ADGB globin domain exhibits atypical nitric oxide-related biochemical properties, including pronounced nitrite reductase activity and unusual heme coordination, consistent with a specialized biochemical role distinct from conventional oxygen transport (17). Beyond canonical oxygen transport and storage, globins are increasingly recognized as versatile signaling proteins involved in nitric oxide metabolism, reactive oxygen species homeostasis, and redox-sensitive cellular signaling pathways (18). Notably, several globins have been reported to exhibit specialized biochemical properties and restricted tissue expression patterns suggestive of regulatory rather than respiratory functions (19, 20, 21). In this context, the unusual multidomain organization and restricted expression profile of ADGB raise the possibility that its globin domain performs specialized regulatory functions within defined cellular environments.

Interestingly, our previous overexpression studies in *Sf9* insect cells revealed the presence of multiple ADGB cleavage products, suggesting that ADGB undergoes post-translational proteolytic processing (22). However, the mechanism regulating this processing, its potential calcium dependence, and its functional significance remained unresolved. Given the presence of a calpain-like domain together with a calmodulin-binding IQ motif within ADGB, we hypothesized that calcium signaling may regulate ADGB proteolysis and potentially modulate the activity or localization of its globin domain.

In the present study, we investigated whether ADGB undergoes calcium-dependent proteolytic processing and examined the biochemical and cellular properties associated with separation of its constituent domains. We show that ADGB undergoes robust calcium-dependent processing that requires cytoplasmic factor(s), while our data argue against CAPN1 and CAPN2 as the principal proteases responsible for this cleavage. CAPNS1 downregulation reduces calcium-dependent ADGB processing, suggesting that CAPNS1 contributes to this process through an as-yet unresolved mechanism. Domain-mapping experiments further identify cleavage regions capable of separating the N-terminal calpain-like and globin-containing regions of ADGB. Notably, the experimentally isolated globin domain displays enhanced interaction with calmodulin and preferential localization to centrosomal structures compared with full-length ADGB. Together, these findings identify calcium-dependent proteolytic processing as a previously unrecognized property of ADGB and raise the possibility that such processing modulates the biochemical accessibility and cellular behavior of its constituent domains.

## Results

### ADGB is proteolyzed in a calcium-dependent manner

We previously reported ADGB to instantaneously produce several smaller products, when exogenously expressed in *Sf*9 insect cells (22), a proteolysis event that has also been described for calpains (9, 23). Similarly, transient overexpression of ADGB in HEK293T cells resulted in the detection of several smaller ADGB products. Following calcium treatment in combination with a calcium ionophore, a characteristic pattern of distinct ADGB cleavage products was observed (Fig. 1*A*). This cleavage pattern was highly robust across independently generated recombinant constructs carrying different tags, including green fluorescent protein (GFP), Glutathione S-transferase (GST), and HaloTag (Fig. S1, *A*–*C*), suggesting that ADGB proteolysis is regulated in a calcium-dependent manner and may be linked to calcium signaling pathways. HEK293T cells were initially selected because their high transfection efficiency provides a tractable system for systematic biochemical characterization of the unusually large ADGB protein and its numerous recombinant constructs. To determine whether the observed processing was restricted to transient expression in this cellular background, we further validated this phenomenon by generating constitutively expressing cellular models, using Clustered Regularly Interspaced Short Palindromic Repeats-based (CRISPR)-based gene knock-in in HEK293T (Fig. 1*B*), HeLa (Fig. S1*D*), and MCF-7 (Fig. S1*E*) cell lines. In all these cell types, characteristic ADGB cleavage products were detected following calcium treatment, indicating that the observed calcium-responsive processing is not restricted to a single cellular model or expression construct. Since ADGB is transcriptionally silent in most cell types (24, 25), we next investigated calcium-mediated ADGB cleavage under more endogenous conditions using CRISPR activation (CRISPRa). Although CRISPRa-mediated induction produced lower overall levels of endogenous ADGB, in line with low endogenous expression levels (25), calcium treatment resulted in detection of a characteristic lower-molecular-weight ADGB product corresponding to the shorter ADGB species generated by the CRISPRa system (Fig. 1*C*). These findings further support the occurrence of calcium-responsive ADGB proteolytic processing. In order to investigate whether ADGB can be cleaved from the release of cytoplasmic Ca^2+^ alone, ADGB-expressing HEK293T cells were lysed without prior calcium treatment, and subjected to further incubation, *in vitro*, at 37 °C. In the absence of EDTA, a divalent cation chelator capable of sequestering Ca^2+^, ADGB underwent rapid cleavage, generating fragment patterns similar to those observed in intact cells. Cleavage occurred immediately upon cell lysis, and the resulting fragments remained stable even after 24 h of incubation *in vitro* (Fig. 1*D*). Collectively, these findings indicate that cytoplasmic Ca^2+^ is sufficient to induce ADGB proteolysis and that this process occurs rapidly and efficiently upon Ca^2+^ exposure.

**Figure 1:**
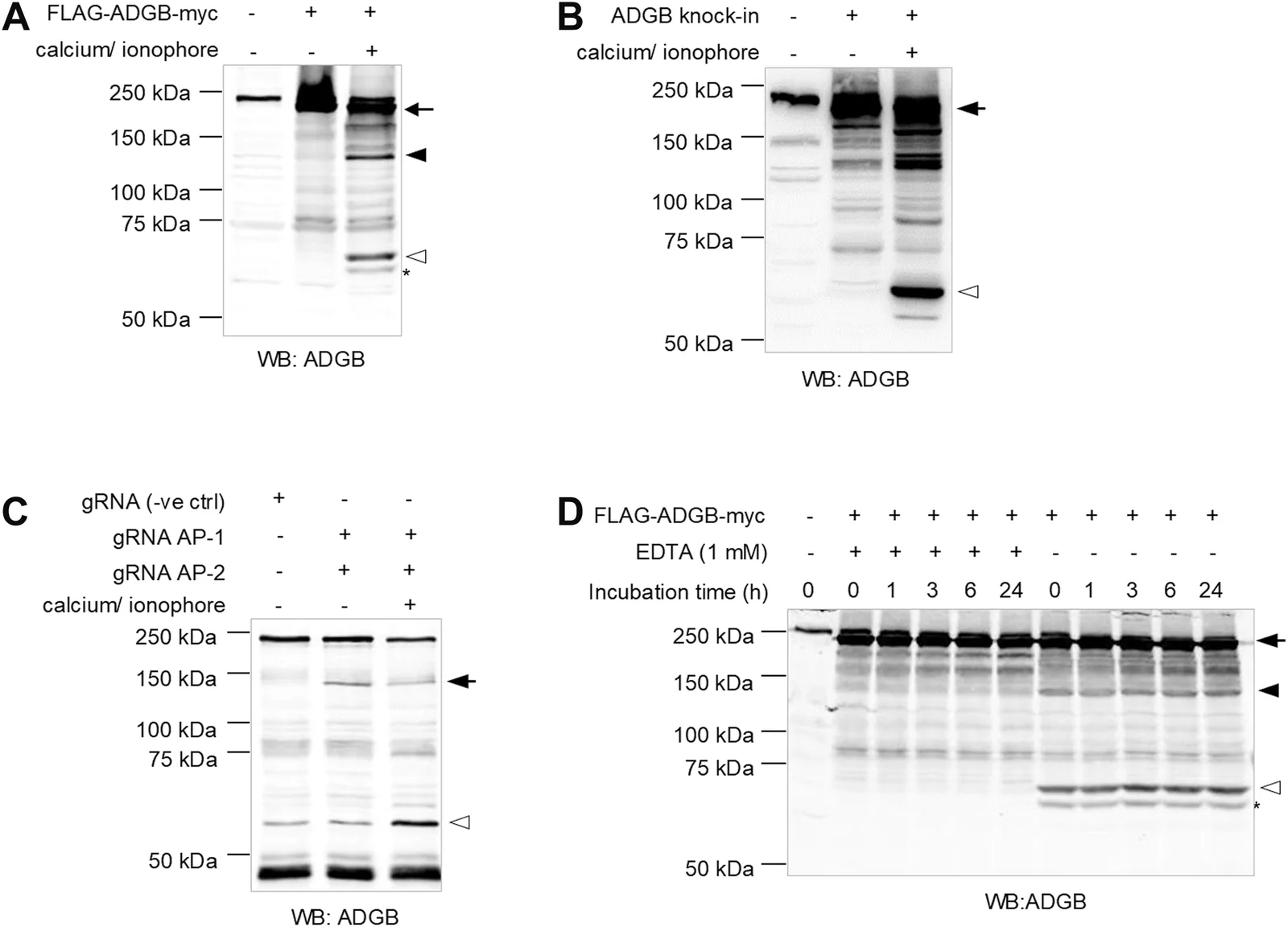
**ADGB exhibits calcium-mediated proteolysis.***A*, FLAG-ADGB-myc was transiently expressed in HEK293T cells with and without the treatment of 50 mM CaCl_2_/25 μM calcium ionophore, and 100 μg of total cell lysate were analyzed by immunoblotting with an antibody against ADGB. Arrows indicate the full length ADGB. Open and closed triangles indicate the characteristic ADGB cleavage detected following calcium treatment. Asterisk indicates a small cleaved ADGB product that is only noticeable with the FLAG-ADGB-myc, which has smaller tags at both N- and C-termini. *B*, calcium-mediated ADGB cleavage is also observed in ADGB knock-in cellular models with constitutive ADGB expression. Full length ADGB is indicated by an arrow. The same cleavage pattern is observed, as indicated by an open triangle. *C*, CRISPRa-induced endogenous ADGB as previously described (Koay *et al.*, 2021) shows a comparable Ca-responsive cleavage pattern following treatment with 50 mM CaCl_2_/25 μM calcium ionophore albeit with a smaller molecular weight which corresponds to the smaller endogenous ADGB produced by the CRISPRa system. Arrow and open triangle indicate the full-length but shorter ADGB and its smaller cleaved product, respectively. *D*, time-course incubation (0–24 h) of total lysates from HEK293T cells transiently expressing FLAG-ADGB-myc, with or without EDTA metal chelator. Sample without EDTA shows rapid cleavage of ADGB upon lysis (0 h), and cleaved products remain stable up to 24 h. Arrow indicates full-length ADGB, while closed triangle, open triangle and asterisk indicate cleaved ADGB products.

### Calcium-mediated ADGB proteolysis is independent of the calmodulin-binding IQ motif

In contrast to classical calpains, ADGB does not contain the canonical calcium-binding penta-EF hand domain, making direct Ca^2+^ binding through such a domain unlikely. ADGB does, however, contain a calmodulin-binding IQ motif embedded within the circularly permutated globin domain (14), raising the possibility that calmodulin could participate in the calcium responsiveness of ADGB processing. To investigate this possibility, we co-overexpressed calmodulin and ADGB in HEK293T cells. In the absence of calcium treatment, the characteristic ADGB cleavage products were not detected following calmodulin overexpression, whereas following calcium treatment a comparable cleavage pattern was observed in the presence or absence of overexpressed calmodulin (Fig. 2*A*). We next inhibited endogenous calmodulin using W-7, a well-established synthetic calmodulin antagonist. The characteristic calcium-responsive ADGB cleavage products remained detectable following W-7 treatment (Fig. 2*B*). These findings suggest that neither potential calmodulin-mediated calcium shuttling nor endogenous cellular calmodulin is required for ADGB proteolysis. To further validate this conclusion, we generated two ADGB variants targeting the IQ motif: an ADGB IQ motif mutant (R918Q/R923Q) unable to bind calmodulin (26), and an ADGBΔIQ mutant lacking the entire IQ-motif. Both constructs displayed calcium-responsive cleavage patterns comparable to those observed for wild-type ADGB (Fig. 2, *C* and *D*). Together, these observations indicate that the IQ motif and calmodulin binding are not required for the calcium-responsive ADGB processing observed under these experimental conditions.

**Figure 2:**
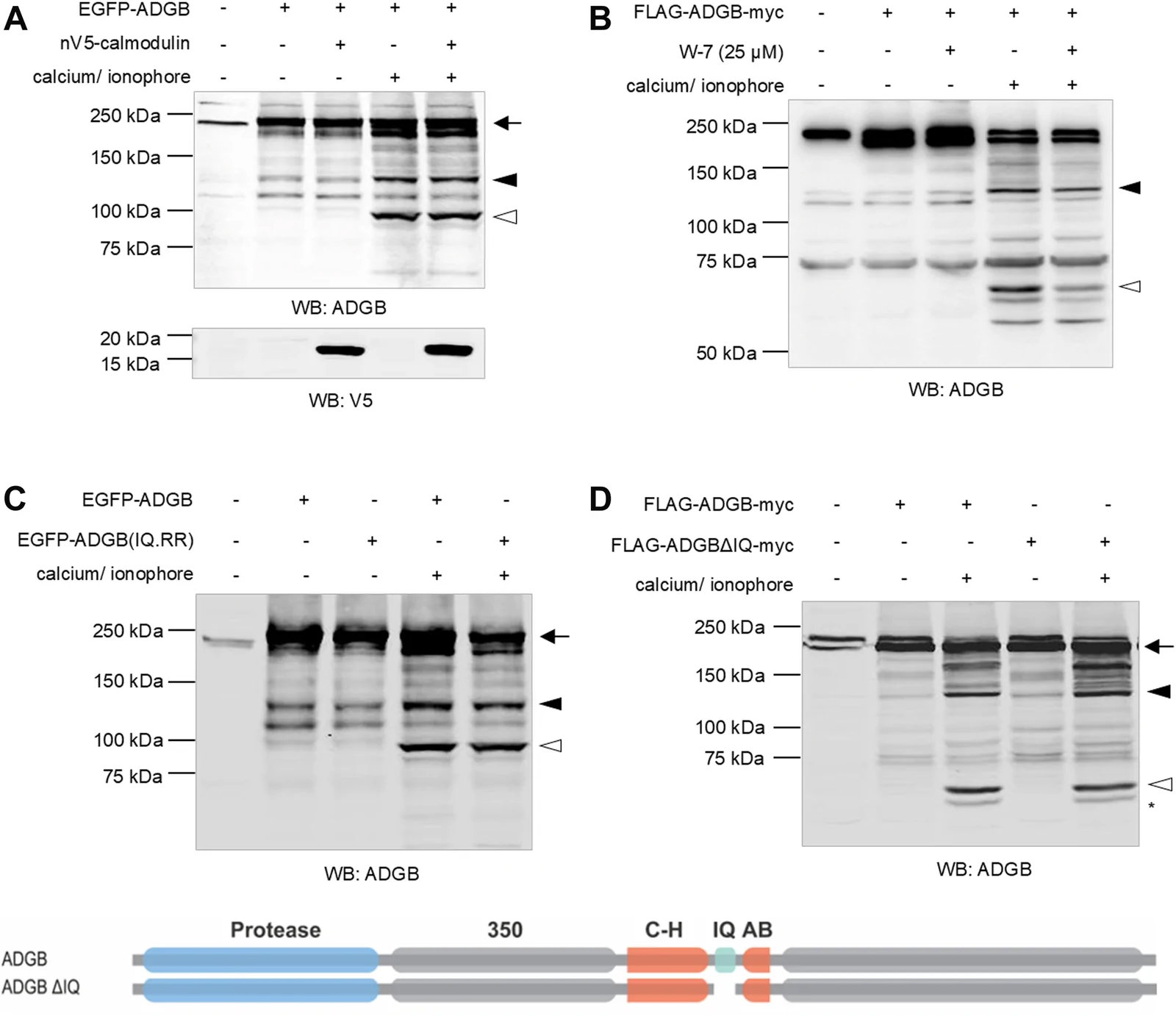
**Calcium-mediated ADGB proteolysis is independent of the calmodulin-binding motif on ADGB.***A*, GFP-ADGB was transiently expressed in HEK293T cells with or without co-expression of V5-calmodulin. Overexpression of V5-calmodulin does not induce the proteolysis of GFP-ADGB in the absence of calcium, and does not promote an increase in ADGB proteolysis in the presence of calcium. Immunoblotting with an antibody against V5 verifies the overexpression of V5-calmodulin. *B*, following pharmacological inhibition of endogenous calmodulin with 25 μM W-7, the characteristic ADGB cleavage products remained detectable following calcium treatment. *C*, R918Q/R923Q ADGB IQ mutant, designed to disrupt the possible binding of calmodulin to the IQ domain (Li *et al.*, 2013) displayed a calcium-responsive cleavage pattern comparable to that observed for EGFP-ADGB (wild type). *D*, following complete deletion of the IQ motif, calcium-responsive ADGB cleavage products remained detectable. Arrows indicate full length ADGB, while closed triangles, open triangles and asterisks indicate ADGB cleavage products.

Given the unusual presence of a globin domain within ADGB, we asked whether this domain contributes to calcium-dependent proteolysis. Deletion of the globin domain did not abolish the characteristic calcium-responsive cleavage pattern, and the observed cleavage products displayed comparable migration under the oxygen conditions examined (Fig. S2). These observations indicate that the globin domain is not required for the calcium-responsive processing characterized here.

### Calcium-dependent ADGB processing involves a cytoplasmic proteolytic activity

In the effort to further dissect the process of calcium-mediated ADGB cleavage, *in vitro* proteolysis assays were carried out using GST-purified recombinant ADGB. Under basal assay conditions, GST-ADGB remained stable and did not undergo spontaneous proteolysis (Fig. S3). However, addition of the cytoplasmic fraction derived from HEK293T cells, but not the nuclear fraction, induced robust GST-ADGB proteolysis, which was further enhanced upon calcium supplementation (Fig. 3*A*). Importantly, the Ca^2+^-dependent increase in GST-ADGB proteolysis was reduced by the addition of BAPTA, a calcium-specific chelator (Fig. 3*B*). These results indicate that a cytoplasmic factor is involved in ADGB proteolysis in a calcium-dependent manner. Fueled by the hypothesis that a calcium-dependent protease might be involved in ADGB cleavage, we next investigated whether members of the calpain protease family play a role in this process. Employing the *in vitro* proteolysis assays, we observed that addition of the pan-calpain inhibitor, calpeptin, can effectively inhibit the calcium-dependent ADGB cleavage in the presence of cytoplasmic proteins (Fig. 4*A*). This result suggests that one or more members of the calpain superfamily might be directly or indirectly involved in the post-translational processing of ADGB under calcium stimulation. Similar results were observed when HEK293T cells transiently expressing ADGB were treated with calpeptin inhibitor, as reflected by a dose-dependent inhibition of calcium-mediated ADGB proteolysis by calpeptin (Fig. 4*B*). A comparable inhibitory effect was observed upon treatment with MG-132, an established proteasome inhibitor known to additionally inhibit calpain activity (Fig. S4*B*). Taken together, these results suggest a possible involvement of one or more members of the calpain family in the proteolysis of ADGB.

**Figure 3:**
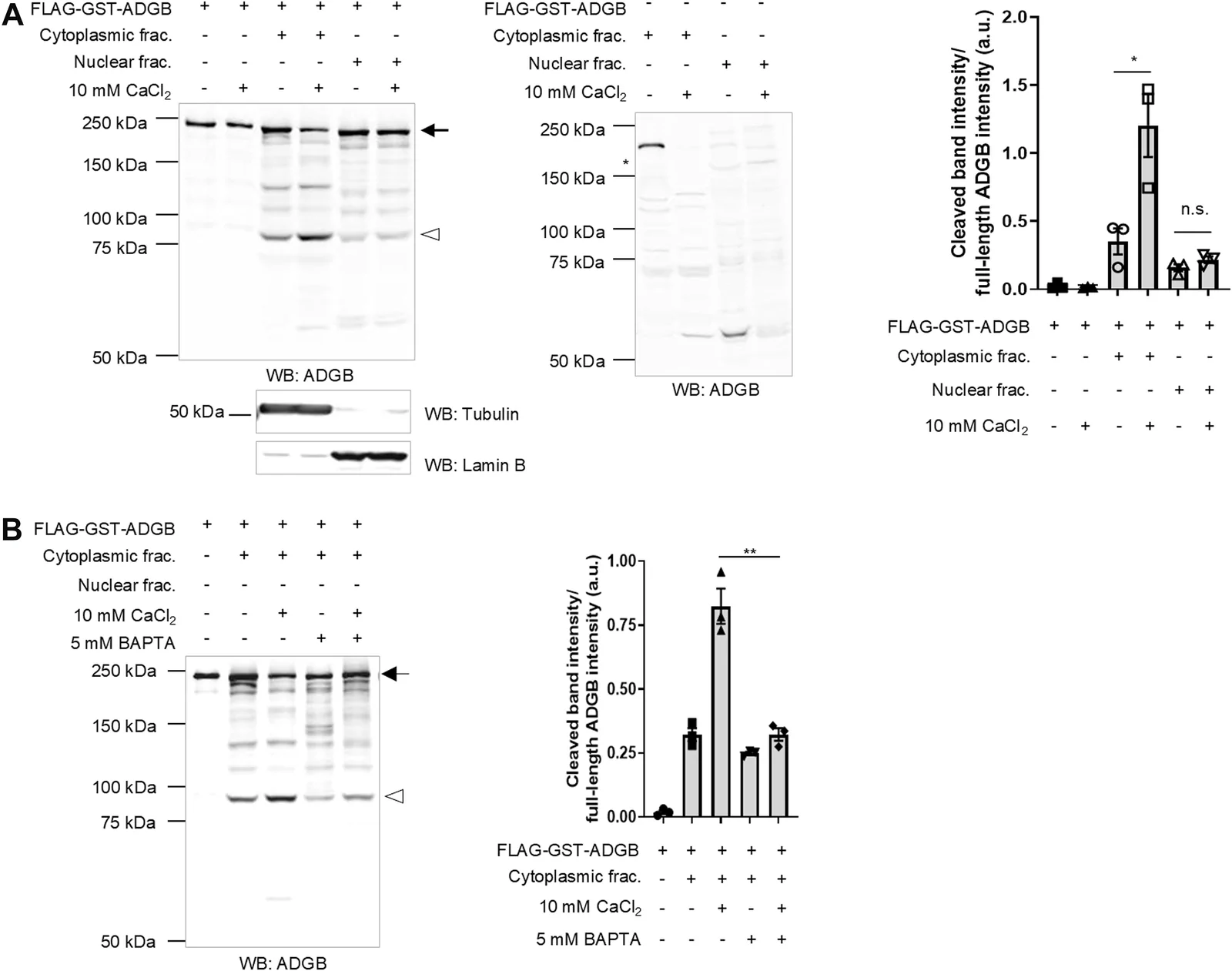
**Calcium dependent cytoplasmic calpains are involved in ADGB proteolysis under calcium signaling.***A*, *in vitro* proteolysis of recombinant ADGB exhibits calcium-dependent cleavage in the presence of cytoplasmic fraction. Purified FLAG-GST-ADGB (∼1.2 μg) was incubated at 37 °C for 3 h, with and without the addition of 35 μg of cytoplasmic or nuclear fraction derived from HEK293T cells, with and without the supplement of 10 mM CaCl_2_. Immunoblot against ADGB shows increased proteolysis of ADGB in cytoplasmic fraction supplemented with 10 mM CaCl_2_. Arrow and open triangle indicate full-length FLAG-GST-ADGB and cleaved product, respectively. Tubulin and lamin B were used as markers for cytoplasmic fraction and nuclear fraction, respectively. A control blot on the right, void of purified FLAG-GST-ADGB shows that the additional bands (marked with *asterisks*) are non-specific bands from the cytoplasmic or nuclear fractions. Quantification of cleaved band intensity over full-length ADGB intensity shows a marked increase in ADGB proteolysis in cytoplasmic fraction supplemented with 10 mM CaCl_2_ (N = 3). *B*, the addition of 5 mM BAPTA (a calcium-specific chelator) to FLAG-GST-ADGB with 35 μg of cytoplasmic fraction, supplemented with 10 mM CaCl_2_ abolished the effect of CaCl_2_ as evident by the immunoblot against ADGB and quantification of cleaved band intensity/full-length ADGB intensity (N = 3). This suggests a calcium-specific activation of ADGB proteolysis in the presence of cytoplasmic proteins.

**Figure 4:**
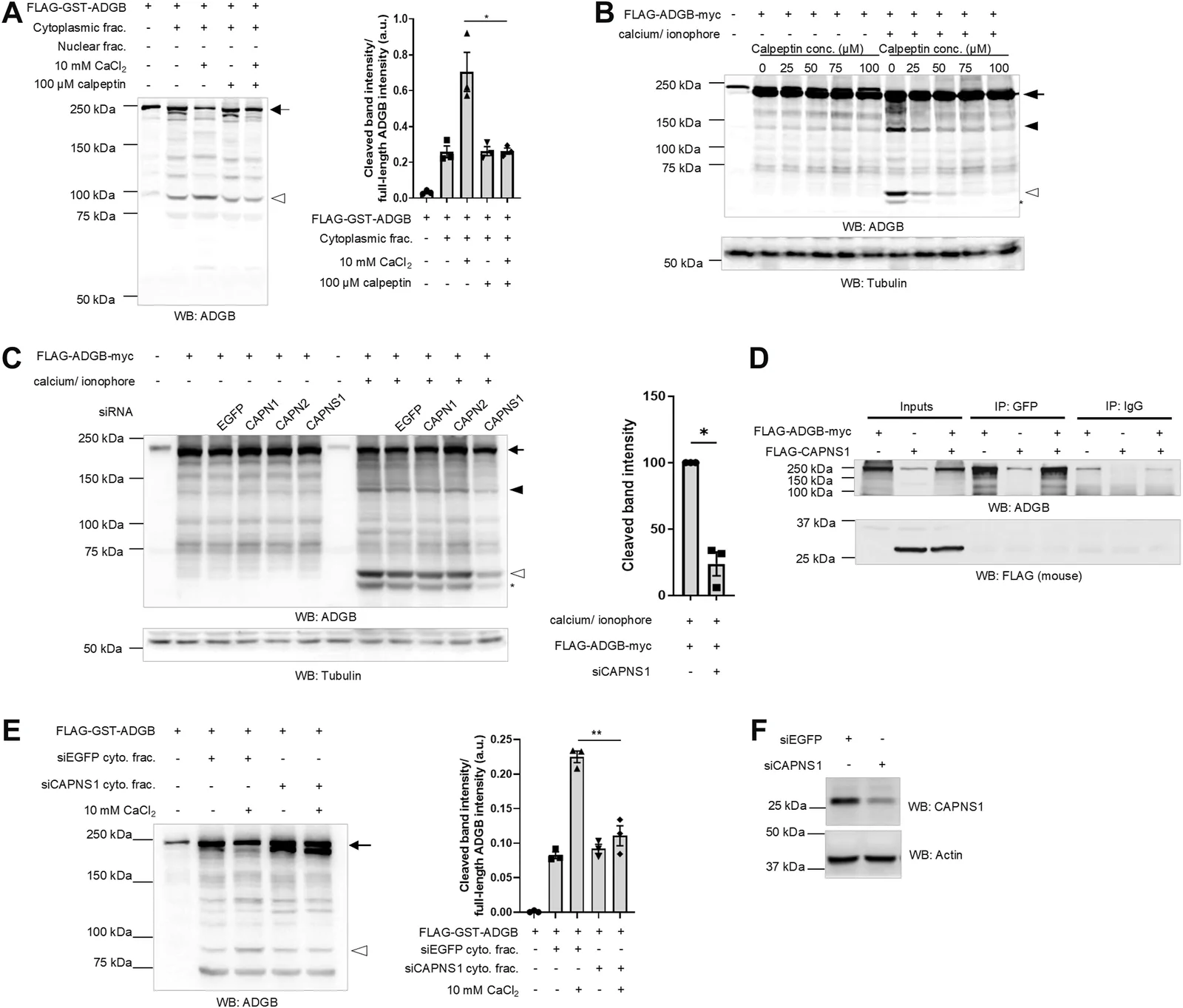
**Calpain smallregulatorysubunit 1 (CAPNS1)downregulationreducescalcium-mediated ADGB cleavagewhereascalpain-1 (CAPN1)orcalpain-2 (CAPN2)downregulation does not.***A*, the addition of 100 μM calpeptin (pan-calpain inhibitor) to FLAG-GST-ADGB with 35 μg of cytoplasmic fraction, supplemented with 10 mM CaCl_2_ abolished the effect of cytoplasmic fraction/Ca^2+^-induced proteolysis of ADGB (N = 3). Quantification of cleaved band intensity/full-length ADGB intensity on the right. Arrow and open triangle indicate full-length and cleaved ADGB products, respectively. *B*, FLAG-ADGB-myc was transiently expressed in HEK293T cells and treated with increasing concentrations of 25 μM, 50 μM, 75 μM, and 100 μM calpeptin for 23 h prior to 1-h treatment with 50 mM CaCl_2_/25 μM calcium ionophore. Immunoblot of cell lysate against ADGB shows a dose-dependent reduction of calcium-mediated proteolysis with calpeptin treatment. Immunoblotting with an antibody against tubulin was used as a loading control. Arrow indicates full-length ADGB, while closed triangle, open triangle and asterisk indicate cleaved ADGB products. *C*, knockdown of EGFP (negative control), CAPN1, CAPN2, and CAPNS1 using gene-specific siRNA pool (MISSION esiRNA, Sigma-Aldrich) shows no effect of CAPN1 and CAPN2 knock-down on calcium-dependent ADGB proteolysis, but CAPNS1 knock-down displays noticeable reduction in ADGB proteolysis, as shown in a representative anti-ADGB immunoblot with anti-tubulin as loading control together with quantification of cleaved band intensity/full-length ADGB intensity specifically for CAPNS1 knock-down (N = 3). Full length ADGB is indicated by arrow, whereas cleaved products are indicated by closed triangle, open triangle, and asterisk. *D*, co-immunoprecipitation of FLAG-ADGB-myc and FLAG-CAPNS1 in HEK293T cells. Recombinant proteins were transiently expressed and cell lysates were then incubated with anti-rabbit IgG (negative control) or anti-ADGB antibody to bind and pull down FLAG-ADGB-myc. The immunoprecipitates were immunoblotted against ADGB and FLAG with 2% cell lysates as input. Immunoblotting with anti-FLAG suggests that CAPNS1 probably has no direct interaction with ADGB. *E*, *in vitro* proteolysis of recombinant ADGB exhibits reduced calcium-dependent cleavage in the siCAPNS1 cytoplasmic fraction *versus* siEGFP cytoplasmic fraction. Purified FLAG-GST-ADGB (∼2.4 μg) was incubated at 37 °C for 3 h, with the addition of 35 μg of cytoplasmic fraction with either CAPNS1- or EGFP- knock-down, with and without the supplement of 10 mM CaCl_2_. Representative immunoblot against ADGB and quantification of cleaved band intensity over full-length ADGB intensity show reduction in proteolysis of ADGB in CAPNS1 knocked-down cytoplasmic fraction supplemented with 10 mM CaCl_2_. Arrow and open triangle indicates full length FLAG-GST-ADGB and cleaved product, respectively. *F*, immunoblotting against CAPNS1 shows reduction in CAPNS1 in siCAPNS1 *versus* siEGFP cytoplasmic fractions.

### Calpain small regulatory subunit (CAPNS1) downregulation reduces calcium-mediated ADGB processing

Given the sensitivity of calcium-dependent ADGB processing to broad protease inhibitors with activity against calpains, we next examined whether the classical calpains CAPN1 and CAPN2 contribute to this process. We first employed the PD150606 inhibitor, with reported specificity towards CAPN1 and CAPN2 (23, 27). In contrast to the robust inhibition observed with the pan-calpain inhibitor calpeptin, treatment with PD150606 did not affect calcium-dependent ADGB proteolysis (Fig. S4*A*), suggesting that CAPN1 and CAPN2 are not the primary mediators of this cleavage event. To verify the effectiveness of PD150606, we employed mammalian-one-hybrid system with a luciferase-based readout. Briefly, presence of the PD150606 inhibitor prevents the catalytic cleavage of a flanked human spectrin (hSpec) cleavage site between a DNA-binding Galactose-responsive transcription factor 4 (Gal4) domain and transcriptional activator virus protein 16 (VP16) domain, by CAPN1 and CAPN2. The intact Gal4-hSpec-VP16 regulates luciferase activity *via* binding and transcriptional activation of a Gal4 response element-driven promoter (Fig. S4*C*). PD150606 effectively inhibited this cleavage-dependent reporter system, confirming that the inhibitor was functionally active in our assays. We next examined the role of CAPN1, CAPN2 and calpain small regulatory subunit (CAPNS1) using siRNA-mediated knockdown in HEK293T cells. Gene knock-down efficiency was verified using RT-qPCR (Fig. S5). Consistent with the results obtained with PD150606, downregulation of CAPN1 or CAPN2 had no detectable effect on calcium-dependent ADGB cleavage. In contrast, CAPNS1 downregulation resulted in a marked reduction in the calcium-mediated cleavage of ADGB (Fig. 4*C*). These findings indicate that CAPNS1 contributes to calcium-dependent ADGB processing, while the absence of comparable effects following CAPN1 or CAPN2 downregulation argues against a straightforward involvement of the classical CAPN1/CAPN2 heterodimers. To determine whether CAPNS1 directly interacts with ADGB, co-immunoprecipitation experiments were performed. No detectable interaction between ADGB and CAPNS1 could be observed, suggesting that CAPNS1 may not regulate this process *via* direct interaction (Fig. 4*D*). In order to further verify the role of CAPNS1 in the proteolysis of ADGB, we performed *in vitro* proteolysis on purified recombinant GST-ADGB using cytoplasmic fractions derived from HEK293T cells subjected to CAPNS1 knock-down, or enhanced green-fluorescent protein (EGFP) knock-down control conditions. In agreement with the cellular experiments, cytoplasmic extracts from CAPNS1-downregulated cells displayed a reduced capacity to mediate calcium-dependent ADGB proteolysis (Fig. 4*E*). Immunoblotting confirmed reduced CAPNS1 protein abundance in the cytoplasmic fractions used for these experiments (Fig. 4*F*), although residual CAPNS1 protein remained detectable following siRNA treatment.

Taken together, these complementary cellular and cell-free experiments indicate that CAPNS1 contributes to calcium-dependent ADGB processing. However, they do not establish a direct role for CAPNS1 in the proteolytic reaction. The mechanism through which CAPNS1 influences ADGB processing, as well as the identity of the protease directly responsible for ADGB cleavage, therefore remain unresolved.

### Calcium-dependent ADGB cleavage might release the globin domain from full-length ADGB chimera

In order to characterize the cleavage pattern of ADGB under calcium stimulation, we analyzed proteolyzed ADGB products using N-terminally tagged constructs combined with immunoblotting against both ADGB and the respective tag proteins. Upon calcium treatment, ADGB reproducibly generated three prominent cleavage products, detectable by ADGB immunoblotting (Fig. 1*A*). To further characterize the calcium-dependent processing of ADGB, we mapped the regions contributing to the observed cleavage products using a series of N-terminally tagged deletion constructs. Immunoblotting against ADGB and the respective N-terminal tags allowed the cleavage products to be distinguished according to their tag composition and apparent molecular weight. For clarity, the two major cleavage products are hereafter referred to as the 135-kDa fragment (closed triangle) and the 60-kDa fragment (open triangle), as shown in Figure 1*A*. Notably, the smallest band marked by an asterisk and the slightly larger but more prominent band marked by open triangle were collectively considered part of the 60-kDa fragment population due to their similar migration shifts across the experimental conditions described below.

To determine the orientation of these cleavage products within the full-length ADGB protein, large GFP or GST tags were introduced at the N-terminus of ADGB. Immunoblot analysis using anti-GFP or anti-GST antibodies demonstrated that the N-terminal tags were retained within the 60-kDa fragment, resulting in the expected proportional increase in apparent molecular weight (∼86 kDa). In contrast, the 135-kDa fragment lacked the N-terminal tags (Fig. 5, *A* and *B*). These results suggest that the 60-kDa fragment originates from the N-terminal portion of ADGB, whereas the 135-kDa fragment belongs to the C-terminal part of ADGB (Fig. S6). To further define the domain composition of these fragments, a series of domain-specific deletion mutants was generated (overview provided in Fig. S7). The strategy relied on predicting size shifts in the cleavage fragments following removal of specific domains. Deletion of the calpain-like protease domain produced corresponding downward shifts in the apparent molecular weight of the smaller cleavage products, whereas the larger cleavage product retained its characteristic migration pattern (Fig. 5, *C* and *D*). Similarly, deletion of the proximal region of the 350-residue uncharacterized domain produced a corresponding downward shift in the apparent molecular weight of the smaller cleavage products (Fig. 5*E*). These findings indicate that both the calpain-like domain and the proximal part of the 350-residue region are present within the 60-kDa proteolysed fragment. Conversely, deletion of the distal region of the 350-residues uncharacterized domain, the globin domain, or either the proximal or distal segments of the 700-residue uncharacterized C-terminal domain, did not appreciably alter the migration of the smaller cleavage products, whereas the larger cleavage product displayed molecular-weight shifts corresponding to the respective deletions (Fig. 5, *F*–*H*, and Fig. S2*B* for globin domain deletion). Together, these migration patterns localize the regions contributing to the different ADGB cleavage products relative to the globin domain. Particularly, these patterns demonstrate that the region from the distal portion of the 350-residue uncharacterized domain toward the end of the ADGB protein is not present in the 60-kDa fragment and is found within the 135-kDa fragment. These findings further indicate that one or more calcium-dependent cleavage regions can be found within the 350-residue uncharacterized domain, a region which is situated C-terminal to the calpain-like protease domain and N-terminal to the globin domain (Fig. S6). Interestingly, transient overexpression of the 700-residue uncharacterized domain truncate also revealed calcium-dependent cleavage within this region (Fig. 5*I*). By employing differential N-terminal and C-terminal tagging of this construct, we could infer the presence of at least two additional regions of calcium-dependent processing within this construct. Since both the 350-residue and 700-residue uncharacterized domains flank the N-terminal and C-terminal part of the globin domain respectively, the presence of calcium-dependent processing within these regions raises the possibility that proteolytic processing could separate the globin-containing region from surrounding portions of full-length ADGB. Finally, in addition to the major cleavage events described previously, we also observed a calcium-dependent processing within the short N-terminal region of ADGB (Fig. S8). Notably, this cleavage pattern resembles the N-terminal processing reported for CAPN1 and CAPN2 (7, 8). Intriguingly, predicted calpain cleavage sites in the ADGB protein overlap with the experimentally inferred cleavage region (Fig. S6), with several high-confidence predicted sites located between residues 550 and 650 (28).

**Figure 5:**
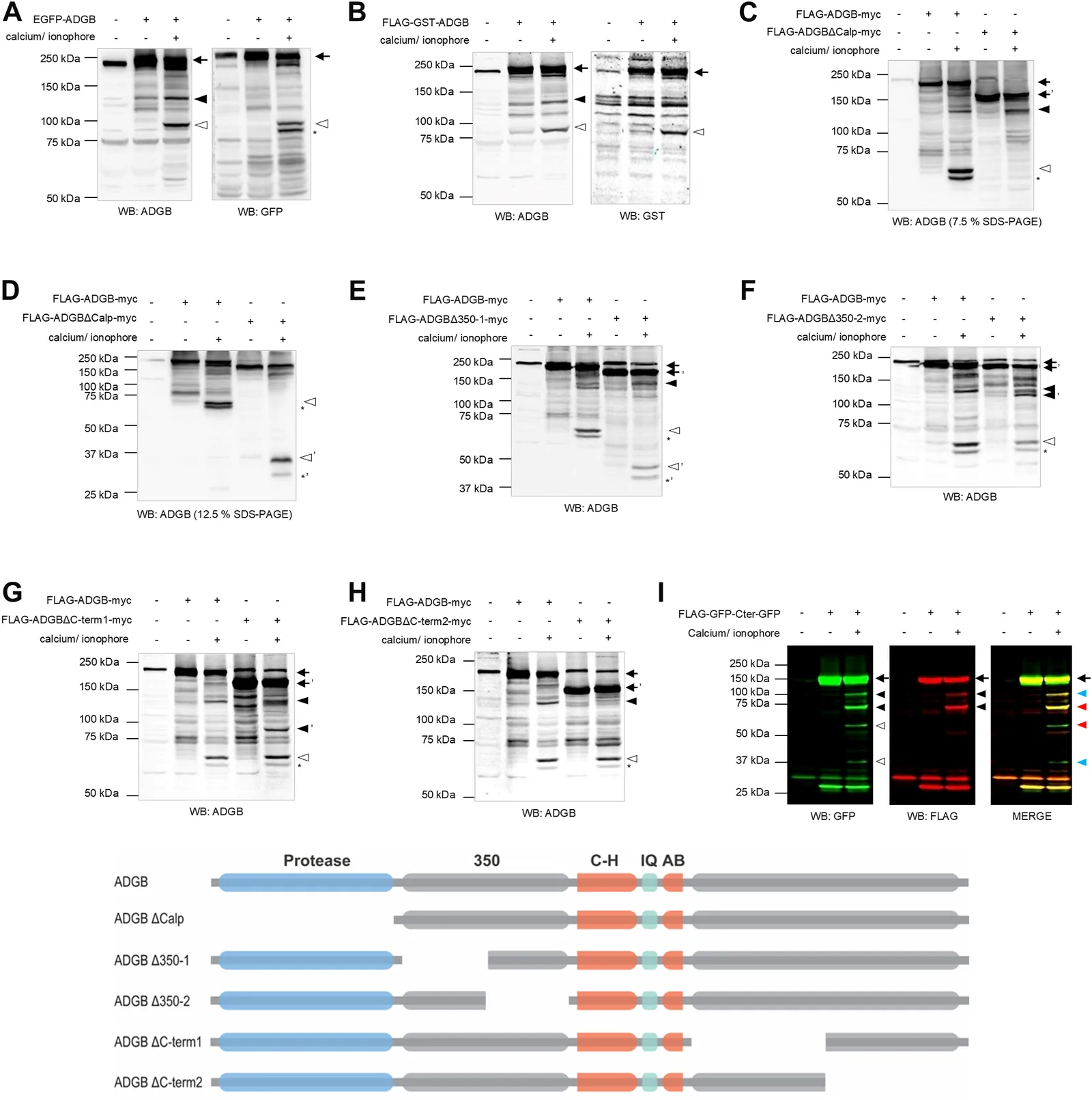
**Mapping of calcium-dependent ADGB cleavage regions using deletion constructs.** GFP-ADGB (*A*) and FLAG-GST-ADGB (*B*) was transiently expressed in HEK293T cells, with and without treatment of 50 mM CaCl_2_/25 μM calcium ionophore, and 100 μg of total cell lysate was analyzed with immunoblot against ADGB and GFP (*A*) or GST (*B*). For GFP-ADGB, the smaller cleaved ADGB products (with calcium treatment), indicated by open triangle and asterisk contain the N-terminus EGFP tag, whereas the larger cleaved ADGB product indicated by closed triangle, does not have the EGFP tag (*A*). Consistently, the smaller cleaved ADGB product of FLAG-GST-ADGB, indicated by open triangle, contains the N-terminus GST tag but not the larger cleaved ADGB product indicated by closed triangle (*B*). Images of immunoblotting against ADGB for GFP-ADGB and FLAG-GST-ADGB in panels A and B are reproduced from Fig. S1, *A* and *B*, respectively. *C–D*, total deletion of the calpain-like protease domain resulted in smaller calcium-responsive cleavage products, with molecular-weight shifts corresponding approximately to the size of the deleted region. Immunoblotting was performed with 7.5% (*C*) and 12.5% (D) SDS-PAGE to facilitate visualization of larger and smaller protein bands, respectively. Deletion of the proximal region of the 350-residue domain (res. 410–588), similarly produced a downward molecular-weight shift of the smaller cleavage products (*E*). The smaller cleaved products are indicated with an open triangle and an asterisk ([’] identify products from the mutant construct). (*F*–*H*) Partial removal of the distal region of the 350-residue domain (res. 641–762) (*F*), or the proximal region of the uncharacterized C-terminal domain (res. 988–1295) (*G*), or the distal region of the uncharacterized C-terminal domain (res. 1296–1667) (*H*), transiently expressed in HEK293T cells with and without calcium treatment. Contrary to (*C*–*E*) and (*F*–*H*), the smaller cleaved products, indicated by open triangle and asterisk ([’] for mutant), displayed migration comparable to WT, whereas the larger cleaved product indicated by closed triangle ([’] for mutant) exhibited molecular-weight shifts corresponding to the respective deletions. *I*, ADGB C-terminal domain flanked with FLAG-GFP at the N-terminus and GFP at the C-terminus were overexpressed in HEK293T cells with and without calcium treatment. Immunoblotting analysis against GFP and FLAG identified calcium-responsive cleavage products retaining either the N- or C-terminal tag. Dual-mode imaging of GFP and FLAG was used to differentiate the N-terminal fragments (with FLAG tag) and the C-terminal fragment (without FLAG tag). Full-length protein is indicated by arrow. The two bands indicated by closed arrow in FLAG WB are of the N-terminal of the truncate, whereas the two bands indicated by open triangle in the GFP WB (void of FLAG tag) made up the C-terminal of the truncate. Based on the molecular weight, it is predicted that the pairs indicated in red or blue, in the merged image, made up the full-length of the ADGB C-terminal truncate, suggesting that at least two calcium-dependent cleavage hotspots are present.

### ADGB globin domain interacts with calmodulin and this interaction is impeded by the C-terminal domain

The presence of an embedded IQ motif within the circularly permuted globin domain prompted us previously to investigate the potential interaction of ADGB with calmodulin (CaM) (29). In our earlier study, however, full-length ADGB did not exhibit a readily detectable interaction with calmodulin. Consistent with these former observations, co-immunoprecipitation of the isolated ADGB globin domain with calmodulin demonstrated robust interaction between these two proteins (Fig. 6*A*). This result indicates that the globin domain within the ADGB chimera might not be able to interact with calmodulin despite its IQ-motif, whereas the isolated globin domain can bind to calmodulin. To further explore this hypothesis, we generated additional fusion constructs. Co-immunoprecipitation experiments using an ADGB construct comprising the N-terminal part of the protein together with the globin domain (residues 1–968) still demonstrated robust interaction with calmodulin (Fig. 6*B*). This suggests that the presence of the region N-terminal to the globin domain does not interfere with calmodulin binding to the IQ-motif. In contrast, an ADGB construct consisting of the globin domain together with the extended C-terminal region (700-residue uncharacterized domain) failed to efficiently interact with calmodulin (Fig. 6*C*). These observations raise a possible mechanistic interference of the extended C-terminal domain on the binding of calmodulin to the IQ-motif within the globin domain and suggest that proteolytic separation may have putative functional consequences. Consistent with this interpretation, a C-terminally GFP-tagged isolated ADGB globin domain, in contrast to its N-terminally tagged counterpart, also failed to efficiently interact with calmodulin (Fig. S9). Altogether, these experiments indicate that the ADGB globin domain, when isolated from the surrounding domains or relieved from C-terminal structural constraints, can interact more effectively with calmodulin.

**Figure 6:**
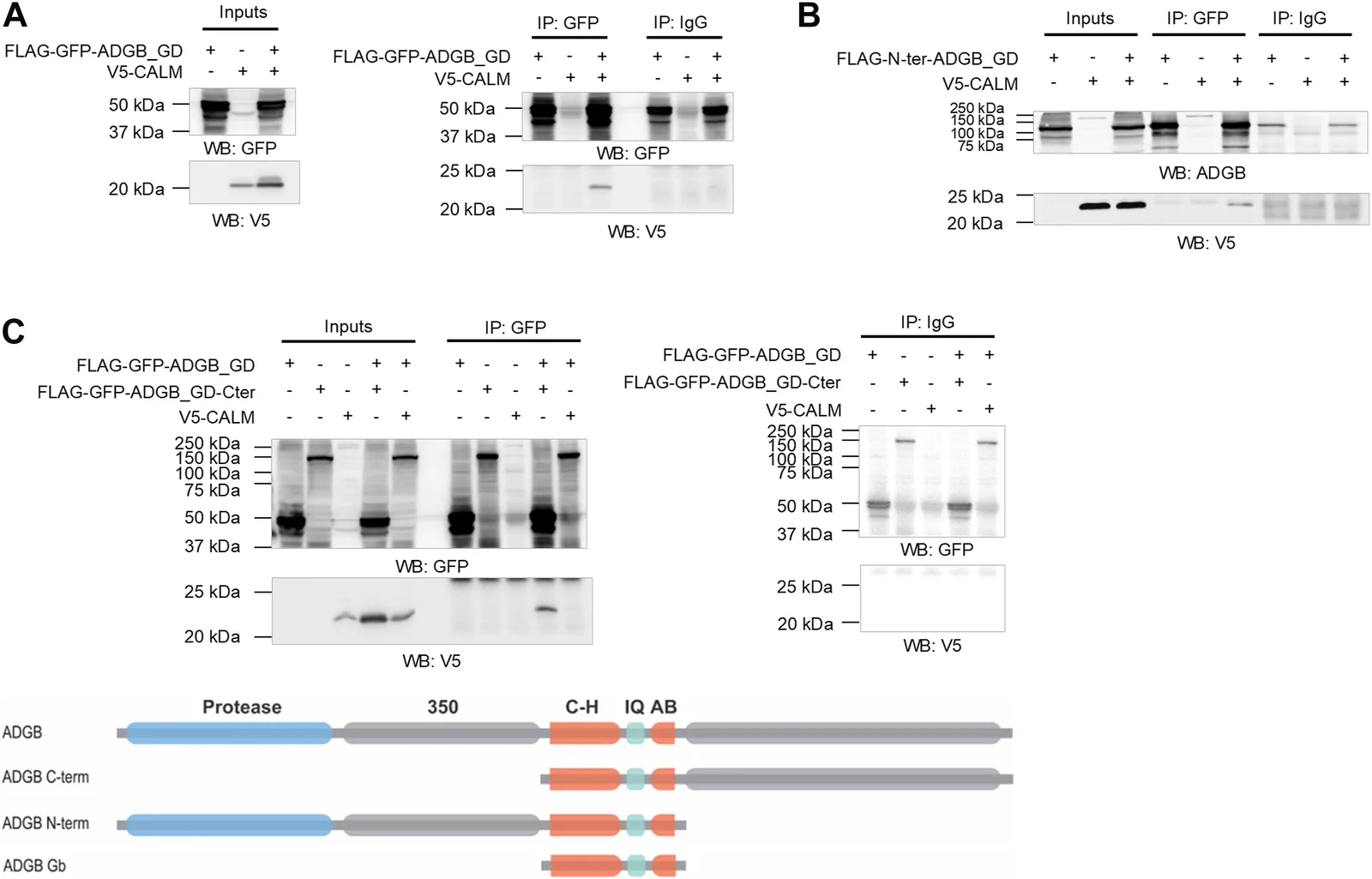
**IQ-motif-containing ADGB globin domain (ADGB_GD) interacts with calmodulin and the interaction isimpededby the long C-terminal domain.***A*, co-immunoprecipitation of FLAG-GFP-ADGB_GD and V5-CALM in HEK293T cells. Recombinant proteins were transiently expressed, and cell lysates were then incubated with anti-rabbit IgG (negative control) or anti-GFP antibody to immunoprecipitate FLAG-GFP-ADGB_GD. The immunoprecipitates were immunoblotted against GFP and V5 with 2% cell lysates as input. V5-CALM was immunoprecipitated with FLAG-GFP-ADGB_GD as shown in the V5 immunoblot. *B*, co-immunoprecipitation of FLAG-Nter-ADGB_GD (without ADGB C-terminal domain) and V5-CALM in HEK293T cells, with protocol as mentioned in (*A*). Immunoblotting against V5 shows no interference in the binding of ADGB_GD in the presence of N-terminal segment and the absence of C-terminal segment. *C*, co-immunoprecipitation of FLAG-GFP-ADGB_GD or FLAG-GFP-ADGB_GD-Cter (without N-terminal segment relative to the globin domain) and V5-CALM in HEK293T cells. The immunoprecipitates indicate that the presence of C-terminal domain (relative to the globin domain) interferes with the binding of V5-CALM onto the ADGB_GD. Immunoprecipitation of FLAG-GFP-ADGB_GD and V5-CALM acts as a positive control.

### The ADGB globin domain preferentially localizes to centrosomal structures

To investigate the significance of ADGB proteolysis in a cellular context, we examined the subcellular localization of different ADGB truncation constructs in HEK293T cells by visualizing recombinant GFP-tagged fusion proteins. We found that the full-length ADGB (GFP-ADGB) displayed a diffuse cytosolic localization pattern throughout the cell (Fig. 7*A*). A similar localization pattern was observed for the ADGB construct consisting of the globin domain together with the long C-terminal domain (GFP-ADGB_GD-Cter, Fig. 7*B*). Intriguingly, ADGB globin domain alone without the long C-terminal (GFP-ADGB_GD, Fig. 7*C*) displayed a distinct dot-like localization, predominantly detected in the perinuclear region. To further characterize these punctate structures, cells were co-stained with the centriole marker pericentriolar material 1 (PCM-1). Immunofluorescence analysis revealed overlaying signals between PCM-1 and the GFP-ADGB_GD puncta (Fig. 7*C*). In contrast, centrosomal localization was only rarely observed for either the full-length ADGB protein or the GFP-ADGB_GD-Cter construct (Fig. 7, *A* and *B*). Quantitative analysis comparing centrosomal *versus* diffuse cytoplasmic localization further demonstrated preferential localization of GFP-ADGB_GD to the centrosome (50.6%) in comparison with the full-length GFP-ADGB and the GFP-ADGB_GD-Cter (Fig. 7*D*). Mutations affecting heme-binding proximal F8 and/or distal E7 residues within the ADGB globin domain did not alter the preferential centrosomal localization, suggesting that this differential localization occurs independently of heme-binding (Fig. S10). To further explore a potential centrosomal association of the ADGB globin domain, we examined endogenous proteins identified by GFP-ADGB_GD co-immunoprecipitation and mass spectrometry (29). Proteins exhibiting log2 enrichment ≥ 1 were analyzed for reported centrosomal or basal body localization using Gene Ontology annotations combined with subsequent Human Protein Atlas-based localization data. The enrichment of 52 endogenous centrosome-associated proteins among GFP-ADGB_GD interactors corroborates the observed centrosomal localization of the isolated globin domain (Table S1).

**Figure 7:**
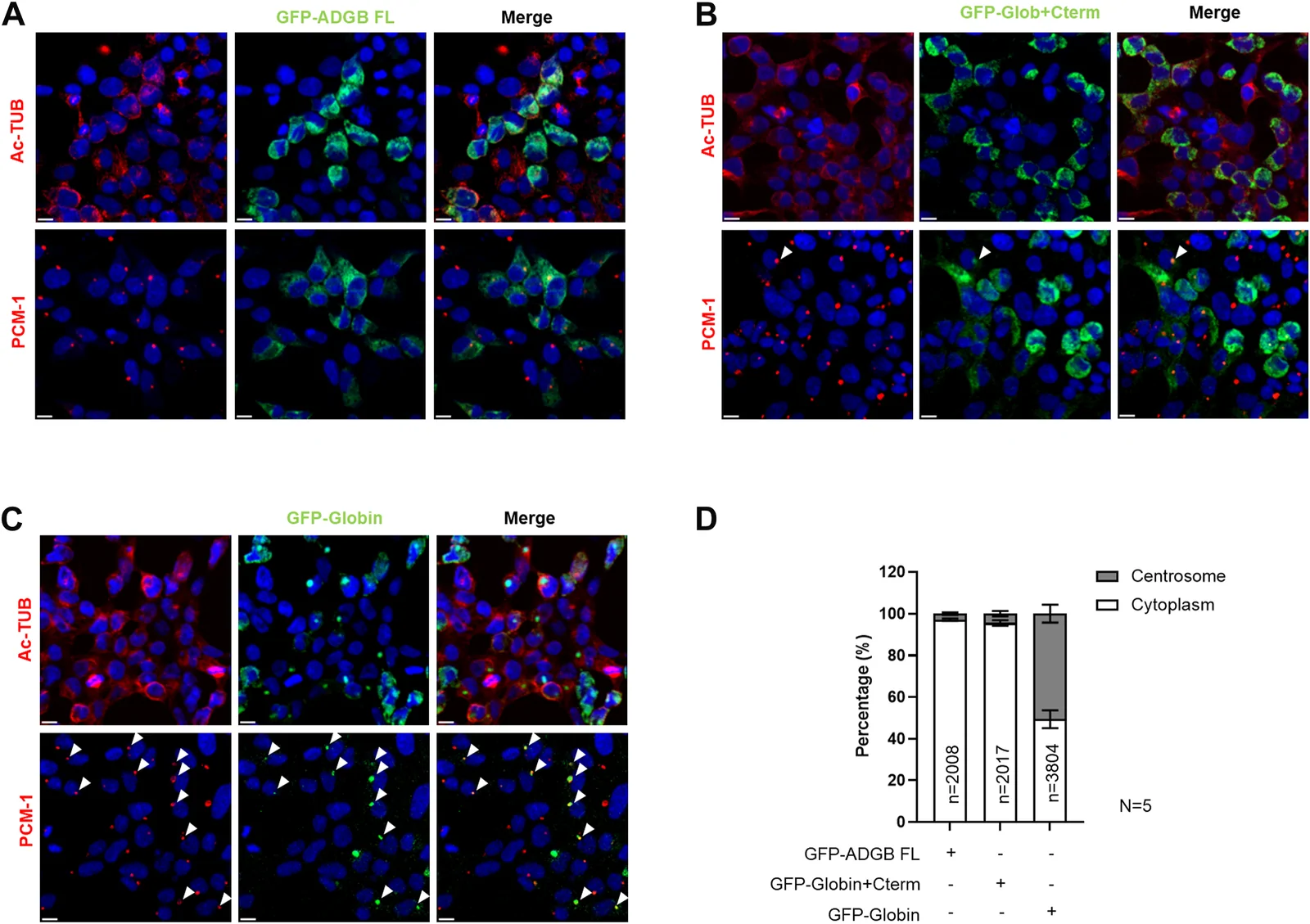
**Recombinant ADGB globin domain preferentially localizes to the centrioles.***A-C*, N-terminally GFP-tagged ADGB FL (GFP-ADGB) (*A*), ADGB globin domain with C-terminal domain (GFP-ADGB_GD-Cter) (*B*), and ADGB globin domain (GFP-ADGB_GD) (*C*) were transiently expressed in HEK293T cells for 24 h prior to 3.7% PFA/PBS fixation. Fixed cells were stained against acetylated tubulin (Ac-TUB) or centriolar marker (PCM-1) prior to mounting with DAPI staining. *Red* signals are the stained Ac-TUB or PCM-1 as indicated, whereas GFP signals show the subcellular localization of ADGB FL and its truncation constructs. GFP-ADGB and GFP-ADGB_GD-Cter are distributed in the cytoplasm, whereas GFP-ADGB_GD shows preferential localization in the centrioles (or centrosome), as evident by the overlaying with PCM-1 signal (*C*). White triangles show the colocalization of GFP signals to the centrosomes. Scale bar represents 10 μm. *D*, statistical analysis of the number of cells displaying localization at the cytoplasm *versus* centrosome. Bar chart represents the average percentages of transfected cells with GFP signals localized at the centrosome *versus* cytoplasm. Average percentages of centrosomally localized GFP-ADGB FL, GFP-ADGB_GD-Cter, and GFP-ADGB_GD are 2.9%, 4.5%, and 50.6%, respectively (n = 5 independent experiments).

Collectively, these findings suggest that ADGB proteolysis may liberate the globin domain from the full-length chimeric protein, thereby enabling its relocalization to the centriole. Given that centrioles serve as precursors of basal bodies during ciliogenesis, these observations suggest a potential functional connection between ADGB proteolytic processing and ciliary biology. A schematic integrating the principal observations and distinguishing experimentally established relationships from proposed consequences of ADGB processing is presented in Figure 8.

**Figure 8:**
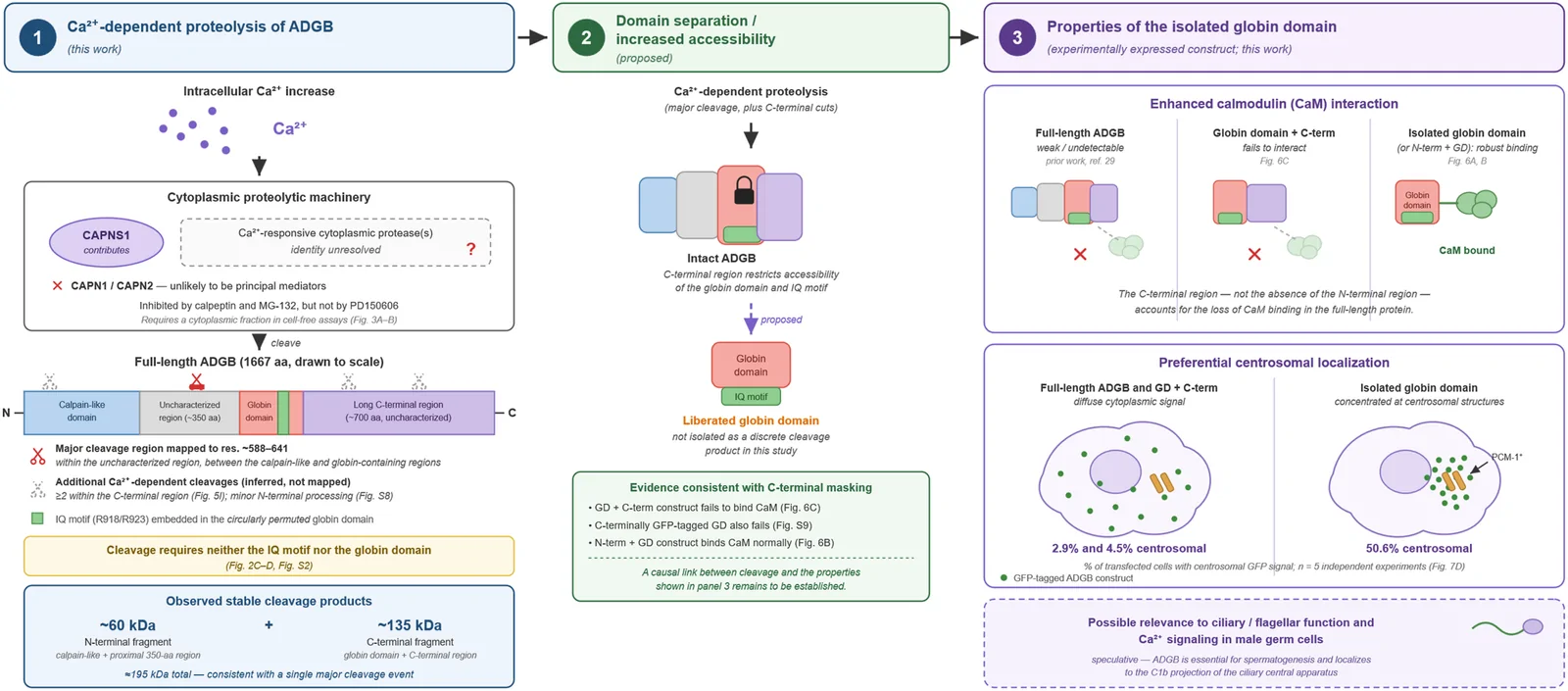
**Working model of calcium-dependent ADGB processing and potential consequences for the globin domain.** (1) Elevation of intracellular Ca^2+^ triggers proteolysis of ADGB by a cytoplasmic, Ca^2+^-responsive proteolytic activity. Cleavage is inhibited by calpeptin and MG-132 but not by PD150606, and is reduced by CAPNS1 downregulation, whereas depletion of CAPN1 or CAPN2 has no detectable effect; the protease directly responsible remains unidentified. The major mapped cleavage region (*red* scissors) lies between residues ∼588 and ∼641, within the uncharacterized region separating the calpain-like and globin-containing halves of the protein, and generates the two stable products of ∼60 kDa (N-terminal) and ∼135 kDa (C-terminal). Additional Ca^2+^-dependent cleavages that were inferred but not mapped (*gray* scissors) include at least two sites within the C-terminal region and a minor N-terminal processing event. Cleavage requires neither the IQ motif nor the globin domain. (2) We propose that processing separates the globin-containing region from the remainder of the chimera and relieves a restriction imposed by the C-terminal region on the accessibility of the globin domain and its embedded IQ motif. A free globin domain was not isolated as a discrete cleavage product in this study, and this step therefore remains hypothetical. (3) Consistent with such a model, the experimentally expressed isolated globin domain, but not full-length ADGB or the globin domain fused to the C-terminal region, interacts robustly with calmodulin and localizes preferentially to centrosomal structures. These constructs were generated by cloning rather than by proteolysis, so a direct causal link between Ca^2+^-dependent cleavage and the altered behavior of the globin domain remains to be established. Domain colors are as in panel 1 throughout.

## Discussion

Post-translational proteolysis represents a fundamental and irreversible protein modification that is often underappreciated and interpreted as protein degradation or turnover. However, limited proteolysis generating stable and functional products is undeniably physiologically relevant (30). Classic examples of cleavage-based enzyme activations include the cleavage of Factor XIII and fibrinogen during blood coagulation (31) and the cleavage of chymotrypsinogen to chymotrypsin in digestion (32). In this study, we report that the chimeric protein ADGB undergoes a limited proteolysis upon treatment with calcium, generating two prominent cleavage products which we termed 60-kDa fragment and 135-kDa fragment for simplicity. Size-based predictions suggest that the 60-kDa and 135-kDa fragments (∼195 kDa) are most probably the products from a single cleavage that separates the full-length ADGB. We comprehensively investigated and showed that the cleavage hotspot is situated between the calpain-like protease domain and the globin domain, with the 135-kDa fragment retaining both the globin domain and the extended C-terminal domain (700-residue uncharacterized domain). The remarkable stability and reproducibility of these cleavage products strongly suggest that ADGB proteolysis represents a regulated post-translational event rather than nonspecific degradation.

The atypical multidomain architecture of ADGB makes this observation particularly intriguing. ADGB contains an N-terminal calpain-like domain fused to a circularly permuted globin domain harboring an embedded IQ motif, followed by an extended C-terminal region of unknown function (14). While ADGB was initially annotated as calpain 16 (CAPN16) or “demi-calpain” (16), the absence of critical catalytic residues within the CysPc domain has long raised uncertainty regarding its intrinsic proteolytic activity. Our findings argue against simple autoproteolysis of ADGB and instead support the involvement of one or more external calcium-responsive cytoplasmic factors. Purified recombinant ADGB remained stable *in vitro* unless cytoplasmic fractions were added, and cleavage was strongly enhanced by calcium and reduced by calcium chelation. Together, these findings indicate that calcium-dependent ADGB processing requires a cytoplasmic component and is unlikely to result solely from intrinsic activity of the ADGB calpain-like domain.

Our pharmacological experiments provide additional evidence for a calcium-responsive cytoplasmic proteolytic mechanism, although the specificity of these observations requires careful consideration. Calpeptin reduced calcium-dependent ADGB processing both in the cell-free assay and in ADGB-expressing cells, while MG-132 produced a comparable inhibitory effect. Because both compounds have relatively broad inhibitory profiles, these findings cannot by themselves identify a particular calpain as the responsible protease. This interpretation is supported by experiments with PD150606, which preferentially inhibits the classical calpains CAPN1 and CAPN2, and by the corresponding siRNA experiments. Neither PD150606 nor depletion of CAPN1 or CAPN2 substantially affected calcium-dependent ADGB processing, arguing against these classical calpains as the principal proteases responsible for the observed cleavage. Thus, while the combined biochemical and pharmacological data support a calcium-responsive cytoplasmic proteolytic mechanism, the identity of the catalytic protease remains unresolved. Other proteolytic pathways therefore cannot be excluded. In particular, although cathepsins are predominantly lysosomal rather than directly calcium-activated cytoplasmic proteases, calcium-dependent changes in lysosomal function can influence their activity and cellular availability. Whether cathepsins or other protease families contribute directly or indirectly to ADGB processing remains to be determined.

Within this context, the effect of CAPNS1 downregulation is particularly intriguing. CAPNS1 is best characterized as the common regulatory subunit required for the stability and activity of the classical CAPN1- and CAPN2-containing calpain heterodimers (33, 34). However, accumulating evidence indicates that the cellular functions associated with CAPNS1 extend beyond a simple structural role within these heterodimers. CAPNS1 deficiency has been linked to alterations in autophagy, cell adhesion, migration, cytoskeletal organization, cell survival, and signaling (35, 36, 37, 38, 39). Importantly, several observations suggest that at least some of these functions cannot be readily explained by the activity of CAPN1 or CAPN2 alone. In fibroblasts, CAPNS1 deficiency markedly impaired traction-force generation, adhesion, and cytoskeletal organization, whereas depletion of CAPN1 or CAPN2 did not reproduce several of these phenotypes (37). CAPNS1 has also been identified as a direct interaction partner of the RasGAP SH3 domain and implicated in the regulation of cell survival and migration (36), supporting the possibility of CAPNS1-dependent protein interactions beyond its canonical association with the classical calpains. Subsequent work linked CAPNS1 to regulation of galectin-3 phosphorylation and secretion independently of CAPN1/CAPN2 catalytic activity (38). Most recently, DeTone *et al.* identified basigin/CD147 as a CAPNS1-interacting protein involved in traction-force generation and provided further evidence for a CAPNS1-dependent function distinguishable from the proteolytic activity of the classical CAPN1/CAPN2 holoenzymes (40). These observations provide relevant precedent for our finding that CAPNS1 depletion reduces calcium-dependent ADGB processing despite the absence of comparable effects following CAPN1 or CAPN2 inhibition or depletion. Importantly, our data do not establish that CAPNS1 directly participates in the proteolytic reaction. Although we did not detect a stable interaction between CAPNS1 and ADGB under our experimental conditions, a transient or indirect association may not be preserved during co-immunoprecipitation. CAPNS1 could therefore influence ADGB processing indirectly, for example, through protein interactions, organization or stability of another proteolytic component, or modulation of the cellular environment in which cleavage occurs. We consequently interpret our findings as indicating that CAPNS1 contributes to calcium-dependent ADGB processing through an as-yet unresolved mechanism, rather than as evidence that CAPNS1 itself, or a CAPN1/CAPN2-containing complex, directly catalyzes ADGB cleavage. Identifying the responsible protease and determining how CAPNS1 influences this process will require further investigation.

The present experiments establish Ca^2+^ responsiveness of ADGB processing but do not define the physiological origin of the calcium signal. Ionomycin was used to elevate intracellular Ca^2+^ independently of a specific calcium-entry pathway, while direct addition of Ca^2+^ reproduced the effect in the cell-free system. Accordingly, the contribution of specific plasma-membrane calcium channels, intracellular calcium stores, or spatially restricted calcium signals to ADGB processing remains to be determined. This question may be particularly relevant in motile ciliated cells, where spatially and temporally regulated calcium signaling contributes to ciliary function.

Our data further raise the possibility that ADGB proteolysis represents an evolutionarily conserved feature of the protein. A recent study in *Tetrahymena thermophila* expressing C-terminally tagged Adgb detected a prominent ∼130-kDa fragment from a 180-kDa full-length protein (41), closely matching the 135-kDa C-terminal cleavage product we identify in the current study. Because the *Tetrahymena* protein lacks a globin domain, this suggests that proteolytic cleavage of ADGB predates the evolutionary acquisition of the globin domain (42), consistent with our finding that the globin domain is dispensable for cleavage.

An intriguing observation of the current study is that isolation of the ADGB globin domain markedly alters its biochemical and cellular properties. While full-length ADGB displayed predominantly diffuse cytoplasmic localization, the isolated globin domain exhibited striking preferential localization to the centrosome/centriole compartment. Similarly, the isolated globin domain displayed enhanced interaction with calmodulin compared with the intact protein. These findings suggest that separation of the globin domain from surrounding ADGB regions alters its structural accessibility, interaction potential, or subcellular targeting. Because our cleavage-mapping experiments identify proteolytic hotspots capable of separating these regions, proteolytic processing provides a potential mechanism through which such domain-specific properties could become accessible. However, a direct causal relationship between calcium-dependent cleavage and globin-domain relocalization remains to be established (20).

Recent structural and biochemical studies considerably expanded our understanding of the unusual ADGB globin domain. Reeder and colleagues demonstrated that the circularly permuted ADGB globin exhibits highly atypical biochemical properties, including unusual heme coordination, pronounced nitrite reductase activity, and formation of five-coordinate NO-heme complexes more characteristic of signaling globins than classical oxygen carriers (17). Structural analyses further revealed unique features of the ADGB globin fold, including stabilization through intramolecular disulfide interactions and replacement of the otherwise highly conserved CD1 phenylalanine residue. Together, these findings strongly support the concept that ADGB functions as a specialized signaling globin rather than a conventional respiratory globin.

Also relevant to the current study, Nie *et al.* recently demonstrated that calmodulin directly interacts with the ADGB globin domain through the embedded IQ motif and enhances its nitrite reductase activity (43). These observations integrate well with our findings. While the IQ motif proved dispensable for calcium-mediated proteolytic cleavage itself, the liberated globin domain displayed enhanced calmodulin interaction and preferential centrosomal localization. One possibility is therefore that calcium-dependent proteolysis represents an upstream regulatory step that exposes or activates distinct biochemical properties of the globin module. In this context, cleavage may dynamically modulate globin accessibility, interaction networks, or signaling capacity within specialized cellular compartments.

The preferential centrosomal localization of the isolated globin domain is particularly intriguing in light of the established association of ADGB with motile ciliogenesis. We previously demonstrated that ADGB is transcriptionally regulated by Forkhead box protein J1 (FOXJ1) and Regulatory factor X2 (RFX2), master regulators of motile ciliogenesis (44). Furthermore, ADGB-deficient mice develop severe defects in spermatogenesis characterized by abnormal sperm head morphology, impaired manchette organization, defective acrosome formation, and complete male infertility, establishing ADGB as an essential factor in specialized flagellated cells (29). The current findings extend this concept by suggesting that calcium-dependent proteolysis may dynamically regulate ADGB localization or function within ciliary and centrosomal compartments. Consistent with this interpretation, ADGB homologs were identified in *T. thermophila* within the central apparatus, a structure critically involved in regulation of dynein activity and ciliary beating in motile cilia (41). More recently, high-resolution structural and proteomic analyses of human motile cilia further localized ADGB to the C1b projection of the central apparatus (45). These observations further strengthen the view that ADGB participates in highly specialized calcium-responsive signaling environments within motile cilia and flagella.

It is well-established that calcium signaling itself plays a central role in ciliary biology. Intraciliary calcium modulates dynein motor activity and ciliary beating, while calmodulin-dependent pathways contribute to centrosomal organization and basal body function (46, 47, 48). In this context, the calcium-triggered liberation of a calmodulin-interacting globin domain with preferential centrosomal localization raises the possibility that ADGB participates in integrating calcium signaling with ciliary regulatory pathways. Although the precise biochemical activity of the liberated globin domain remains unresolved, the combined findings support a model in which ADGB functions as a dynamically regulated signaling protein whose proteolytic processing modifies its intracellular localization and interaction landscape.

A notable aspect of the current study is that the mechanistic and localization analyses were primarily performed in HEK293T cells using ectopic expression systems. These approaches were intentionally chosen to enable controlled biochemical dissection of ADGB proteolysis and systematic analysis of multiple truncation and tagging constructs of this unusually large multidomain protein. Given the very low endogenous expression of ADGB outside specialized ciliated and flagellated cell types (25), such systems provide a tractable approach for investigating ADGB processing and domain-specific properties. Importantly, the observed calcium-dependent processing was not restricted to a single construct or cellular background: it was reproduced using independently generated ADGB constructs carrying different tags, in CRISPR knock-in models in HEK293T, HeLa, and MCF-7 cells (2 additional cell lines with low endogenous ADGB expression levels (24)), following CRISPR-mediated activation of endogenous ADGB, and in complementary cell-free proteolysis assays. This convergence across independent experimental approaches supports the robustness of the observed processing phenomenon. Nevertheless, establishing its physiological relevance will require analysis of endogenous ADGB in differentiated motile ciliated cells and *in vivo*. Endogenous tagging and advanced imaging approaches will be particularly valuable for defining the temporal dynamics, cellular specificity, and physiological consequences of ADGB processing in its native context.

In conclusion, our study identifies calcium-dependent proteolytic processing as a previously unrecognized property of ADGB. Complementary cellular and cell-free experiments indicate that this processing requires a calcium-responsive cytoplasmic component and is unlikely to be mediated primarily by the classical calpains CAPN1 or CAPN2. CAPNS1 depletion consistently reduced ADGB processing, indicating that CAPNS1 contributes to this process through an as-yet unresolved mechanism, while the identity of the catalytic protease remains unknown. Domain-mapping experiments further indicate that proteolysis can separate major functional regions of the ADGB chimera. Notably, the experimentally isolated globin domain displays enhanced interaction with calmodulin and preferential localization to centrosomal structures compared with full-length ADGB. Although a direct causal relationship between calcium-dependent cleavage and these altered properties remains to be established, our findings raise the possibility that proteolytic processing modulates the biochemical accessibility and subcellular behavior of individual ADGB domains. Together with the established association of ADGB with motile cilia and flagella, these observations provide a framework for investigating how calcium-responsive processing contributes to the regulation and physiological function of this unusual multidomain globin.

## Experimental procedures

### Cloning of recombinant human *ADGB* gene and the construction of ADGB mutants

Full-length human ADGB and fragments of the gene were amplified from pLenti6-nGFP-ADGB for the generation of ADGB full length, deletion mutants or truncates. All plasmids were cloned into pFLAG-CMV-6a expression vector (Sigma-Aldrich), unless otherwise indicated. An overview of mutant constructs can be found in Figure S7. Detailed descriptions of the cloning protocol can be found in the supplementary methods. All ligation reactions was carried out in temperature-cycle fashion in a thermal cycler programmed for 8 h to cycle between 30 s at 10 °C and 30 s at 30 °C with 12 cycles per hour (modified from (49)), following heat-inactivation at 65 °C for 20 min. Ligated plasmids were transformed into DH5-α (*Escherichia coli*) chemically competent cells. All plasmid constructs generated, modified, or received from external sources were verified by Sanger sequencing prior to experimental use.

### HEK293T cell culture, transfection, and RNAi

HEK293T cells were cultured and maintained in Dulbecco's Modified Eagle Medium (DMEM) (ThermoFisher) medium supplemented with 10% fetal bovine serum (FBS) (Chemie Brunschwig) and penicillin-streptomycin mixed solution (PS, penicillin: 100 units mL^−1^, streptomycin: 100 μg mL^−1^, ThermoFisher), DMEM/FBS/PS for simplification, and incubated at 37 °C in a humidified incubator with 5% CO_2_. Unless otherwise stated, all transient expressions in this study were carried out with the calcium-phosphate precipitation method of transfection. Prior to transfection, HEK293T cells were seeded on 6-well plate(s) at a density of 5.2 × 10^4^ cells per cm^2^ of the dish surface area and were allowed to grow at 37 °C/5% CO_2_ for 24 h. At 60% - 80% confluency, medium was aspirated and replaced with DMEM/FBS/PS containing 25 μM chloroquine (Sigma-Aldrich). In each well, 10% (v/v) of transfection mixture was added to the medium, consisting of 50% (v/v) 1.2 μg plasmid DNA/250 mM CaCl_2_ and 50% (v/v) HBS buffer (50 mM HEPES, 280 mM NaCl, 10 mM KCl, 1.5 mM Na_2_HPO_4_, 12 mM glucose). Transfected cells were incubated at 37 °C/5% CO_2_ for 24 h before the medium was replaced with fresh DMEM/FBS. Cells were harvested 48 h post-transfection. For gene knock-down experiments, the same protocol was used with the addition of 100 pmol of esiEGFP/esiCAPN1/esiCAPN2/esiCAPNS1 (Sigma-Aldrich) in a 6-well format, with or without the addition of plasmid DNA.

### Experimental hypoxia and oxidative stress

Hypoxia in cultured cells was induced by incubating the cells in a hypoxia chamber (Baker Ruskinn, InvivO_2_ 400) with a controlled environment of 0.2% O_2_, 5.0% CO_2_, 60% air humidity, at 37 °C. For transient overexpression experiments, transfected HEK293T cells were initially incubated under normoxic conditions at 37 °C/5% CO_2_ for 24 h post-transfection to allow for cell recovery and protein expression, after which the cells were deprived of oxygen by incubation in the hypoxia incubator chamber. Cells were maintained in hypoxic environment for 24 h prior to harvesting. For oxidative stress induction, HEK293T cells that were pre-incubated 24 h in the hypoxia chamber were transferred and incubated under normoxic conditions for 1 h prior to harvesting, in order to stimulate the production of reactive oxygen species. Validation of hypoxia and reoxygenation was done through detection and subsequent lack of detection of HIF-1α *via* immunoblotting.

### Treatment with calcium and pharmacological inhibitors

Calcium-stimulated ADGB proteolysis was carried out by treating the transfected HEK293T cells with 50 mM CaCl_2_ alongside with 25 μM calcium ionophore A23187 (Sigma-Aldrich) for 1 h prior to cell lysis. Pharmacological intervention of calcium-mediated ADGB proteolysis was carried out by treating the cells with inhibitors W-7 (Brunschwig, at 25 μM), MG132 (10 μM), calpeptin (Sigma-Aldrich, 25–100 μM), or PD150606 (Sigma-Aldrich, 5–25 μM) at the indicated concentrations for 23 h prior to CaCl_2_/calcium ionophore treatment.

### dCas9-VPR-mediated expression of endogenous ADGB in HEK293T cells

The transcriptionally suppressed or silent *ADGB* gene in HEK293T cells were activated by employing the nuclease-null-Cas9 (of *Streptococcus pyogene*) with tandem fusion of VP64-p65-Rta tripartite activator (dCas9-VPR) (50) as described earlier (24) along with *ADGB* promoter-targeting guide RNAs (gRNAs). *ADGB* promoter-targeting gRNAs (gRNA1, 5′- GCTCTCCGGGCGCTGGACGC -3'; gRNA2, 5′- TTGCGTCCCTCTGCAGCCAC -3′) were cloned into and expressed from pSPgRNA plasmid (Addgene #47108). For dCas9-VPR activation of *ADGB* gene expression, HEK293T cells were transfected using Roti-Fect (ROTH) method of plasmid DNA delivery, according to the manufacturer’s instruction. Cells were transfected in 6-well plates, seeded with 5.2 × 10^4^ cells per cm^2^ surface area, 24 h prior to transfection. In each well, 1125 ng of dCas9-VPR was delivered together with 375 ng of each gRNA1 and gRNA2. Cells were grown for 24 h before the transfection medium was replaced with fresh DMEM/FBS/PS and allowed to grow for another 24 h before CaCl_2_/calcium ionophore treatment, as described above.

### RNA extraction and quantitative PCR

Total cellular RNA was extracted as previously described (51). Total RNA (2 μg) was reverse transcribed (RT) using the PrimeScript RT reagent kit (Takara Bio) and cDNA levels were estimated by quantitative polymerase chain reaction (qPCR) using the primers listed in Table S2 and a KAPA SYBR FAST qPCR reagent kit (Sigma-Aldrich) in a CFX96 C1000 Thermal Cycler (BioRad). Transcript levels were calculated as described before (52) and displayed as relative expression levels.

### Cell lysis and immunoblotting

For protein extraction, cells were gently washed with ice-cold phosphate-buffer-saline (PBS) buffer and a non-denaturing lysis buffer (10 mM Tris, pH 8.0, 1 mM EDTA, 400 mM NaCl, 0.1% NP-40, 2 μg mL^−1^ leupeptin, 2 μg mL^−1^ pepstatin, 2 μg mL^−1^ aprotinin, 1 mM PMSF) was added, cells were scraped, transferred to 1.5 ml microcentrifuge tubes and lysed on ice for 10 min. The lysate was centrifuged at 14,000 RPM, 4 °C for 10 min. The concentration of the soluble protein fraction was measured using Bradford (reagent from Brunschwig) protein assay as described previously (53).

Soluble proteins were separated with sample loading buffer (50 mM Tris-HCl, pH 6.8, 2% (w/v) SDS, 10% (v/v) glycerol, 100 mM DTT, 0.004% (w/v) bromophenol blue) in denaturing resolving gels (83 mm × 50 mm × 1.5 mm) consisting of linear 7.5%, 10.0% or 12.5% (w/v) polyacrylamide/N,N′-methylene-bis-acrylamide (AppliChem, 37.5:1), 375 mM Tris-HCl, pH 8.8, and 0.1% (w/v) SDS. The separated proteins were subsequently transferred onto nitrocellulose membrane (Amersham Protran Western blotting membranes) in Towbin buffer (25 mM Tris-HCl, 192 mM glycine, 20% (v/v) ethanol) at 100V for 2 h. After transfer, the membrane was blocked for 1 h with 5% (w/v) skim milk in TBST buffer (25 mM Tris-HCl, pH 7.4, 150 mM NaCl, 0.1% Tween-20) at room temperature, followed by overnight incubation at 4 °C with primary antibodies in 1% skim milk/TBST. The membrane was then washed three times with TBST buffer (8 min each), followed by incubation with secondary antibody (Sigma-Aldrich), for 1 h at room temperature and washed for three times with TBST (8 min each). The membrane was dried and fluorescent signals were visualized Li-COR Odyssey infrared imaging system as described previously (54). A list of antibodies is found in the supplementary methods.

### Purification of FLAG-GST-tagged ADGB

FLAG-GST-ADGB was produced in HEK293T cells for purification. HEK293T cells were cultured on 15-cm dishes and transfected using the calcium phosphate precipitation method as described above. In each 15-cm dish, 20 μg of pFLAG-GST-ADGB plasmid was transfected and the cells were harvested 48 h post-transfection for purification. Culture medium was aspirated and the cells were gentle washed with ice-cold PBS to remove excess medium. Triton-X lysis buffer (20 mM HEPES-NaOH, pH 7.5, 3 mM MgCl_2_, 100 mM NaCl, 1 mM EDTA, 1% Triton-X, 1 mM DTT, 1 mM PMSF) was then added to the dish and the cells were scraped and transferred into micro-centrifuge tubes, and incubated on ice for 10 min to allow cell lysis. Cell lysate was centrifuged at 14,000 RPM, 4 °C for 10 min. The supernatant was filtered through 0.45-μm filter and incubated with Glutathione Sepharose 4B (GSH4B) (GE Healthcare) (pre-washed with Triton-X lysis buffer) for 90 min at 4 °C with rotation. After the incubation, GSH4B resin was centrifuged at 2000 RPM, 4 °C for 2 min, and the supernatant was discarded. The resin was washed with Washing buffer (20 mM Tris, pH 8.0, 200 mM NaCl, 1 mM EDTA, 1 mM DTT, 2 mM ATP, 10 mM MgSO_4_) for 4 times and once with TED buffer (20 mM Tris, 1 mM EDTA, 1 mM DTT). Bound FLAG-GST-ADGB was released from the resin by incubation with Elution buffer (50 mM Tris, pH7.5, 1 mM EDTA, 100 mM NaCl, 0.1% Triton-X, 80 mM Glutathione) at 4 °C for 2 h, with rotation. Following centrifugation at 2000 RPM, 4 °C for 2 min, the eluted protein was recovered in the supernatant and dialysed in TED buffer using dialysis kit (GE Healthcare) at 4 °C for 20 h. Purified FLAG-GST-ADGB was collected, and the protein concentration was measured by Bradford protein assay. Purified protein was snap-frozen in liquid nitrogen and stored at −80 °C until use.

### Extraction of cytoplasmic and nuclear fractions

Cytoplasmic and nuclear fractions were isolated from HEK293T cells with a protocol adapted from (55). HEK293T cells were cultured on 15-cm dishes until confluency. After the removal of medium and gentle washing with ice-cold PBS, cells were lysed in Lysis buffer A (10 mM HEPES. pH 7.4, 10 mM KCl, 1.5 mM MgCl_2_, 0.5% NP-40, 1 mM DTT, 1 mM PMSF). Cells were scraped in Lysis buffer A, transferred to a centrifuge tube, and incubated on ice for 20 min. The cell lysate was centrifuged at 14,000 RPM, 4 °C for 10 min and the supernatant was recovered as the cytoplasmic fraction. The pellet was then resuspended in Lysis buffer B (20 mM HEPES, pH 7.9, 400 mM NaCl, 1.5 mM MgCl_2_, 0.2 mM EDTA, 15% glycerol, 1 mM DTT, 1 mM PMSF) and incubated at 4 °C for 20 min with rotation. The resuspended pellet was sonicated and centrifuged at 14,000 RPM, 4 °C for 10 min. The supernatant recovered as the nuclear fraction. To assess the purity of each fraction, 50 μg were resolved on a 7.5% denaturing polyacrylamide gel and immunoblotted against tubulin (cytoplasmic content) and lamin B (nuclear content).

### *In vitro* cleavage of ADGB

For *in vitro* cleavage of ADGB in cells lysate, HEK293T cells transiently expressing FLAG-ADGB-myc or EGFP-ADGB were lysed in non-denaturing lysis buffer with or without the chelating agent EDTA. 100 μg of total protein lysate was incubated at 37 °C for 0, 1, 3, 6, or 24 h. The reaction was stopped by adding 5X sample loading buffer and heating to 95 °C for 5 min.

For *in vitro* cleavage of purified ADGB, purified FLAG-GST-ADGB (∼1.2 μg) was reconstituted in TED buffer and incubated at 37 °C for 22 h. To examine the effect of calcium ions on ADGB cleavage, purified FLAG-GST-ADGB was incubated with or without 10 mM CaCl_2_. Subsequently, FLAG-GST-ADGB was also incubated with 35 μg of cytoplasmic or nuclear extract, with and without the addition of 10 mM CaCl_2_. Similarly, all reactions were stopped by adding 5X sample loading buffer and heating to 95 °C for 5 min. The presence of cleaved ADGB was determined by immunoblotting against ADGB.

### Immunofluorescence and microscopy

To investigate the localization of full-length ADGB and its truncated mutants, plasmid DNA coding for the respective GFP-tagged proteins was overexpressed in HEK293T cells cultured on glass coverslips, using the calcium phosphate precipitation method as described above. Cells were fixed with 3.7% paraformaldehyde/PBS for 10 min at RT 48 h post-transfection. The fixing solution was completely removed, followed by washing with 3 ml of PBS solution. Then, cells were permeabilized with 1 ml of 10% Triton-X/PBS, on ice for 15 min and blocked with 1 ml of 10% FBS/PBS at room temperature for 1 h. Subsequently, the 10% FBS/PBS was removed and the cells were washed with 3 ml of PBS solution for 1 h. The cells were then incubated overnight with mouse anti-acetylated tubulin (Santa Cruz) or anti-PCM-1 (Proteintech) diluted at 1/200 in 10% FBS/PBS. After overnight incubation, cells were washed in ice-cold PBS for 1 h, followed by incubation with secondary Alexa Fluor goat anti-mouse or anti-rabbit IgG (Invitrogen) diluted 1/1000 in PBS. Cells were again washed in PBS for 1 h and carefully mounted on a microscope slide with a Fluoromount-G, with DAPI (SouthernBiotech). The fluorescent signals were detected and imaged on a Nikon Eclipse fluorescent microscope (Nikon Corporation) or Leica SP5 confocal microscope (Leica Microsystem).

### Mammalian one-hybrid system

In order to measure the potential proteolytic activity of ubiquitous calpains, notably CAPN1 and CAPN2, the mammalian one-hybrid system was employed. In this system, a short peptide containing the calpain proteolytic cleavage site was cloned between a Gal4 DNA-binding domain and VP16 transcriptional activator domain. Employing luciferase-based assays, Gal4-VP16 fusion protein binds on concatemerized Gal4 responsive element-driven E1b promoter-dependent firefly luciferase gene and increases the luciferase signal. In contrast, the separation of Gal4 and VP16 domains *via* proteolytic cleavage by calpains results in a diminished luciferase signal. Here, the human spectrin (hSpec) cleavage site was cloned between Gal4 and VP16 (see supplementary methods).

HEK293T cells were seeded with 5.2 × 10^4^ cells per cm^2^ surface area 24 h prior to transfection using calcium phosphate precipitation method of plasmid DNA delivery, as described above. After 24 h of incubation, medium was aspirated and replaced with DMEM/FBS without antibiotics. In this experiment, cells were transfected with 250 ng of pGRE5xE1b, 250 ng of pM3-Gal4-hSpec-VP16, along with 5 ng of pRL-SV40 *Renilla* luciferase control plasmid. Cells were treated with 5 μM, 10 μM, and 25 μM of PD150606, 6 h after transfection, with subsequent incubation for 16 h. Cells were then lysed with Passive Lysis Buffer (Promega), and the luciferase activities of soluble lysate were measured using the Dual Luciferase Reporter Assay System as previously reported (54).

### Generation of ADGB knock-in cell models

CRISPR-based *ADGB* knock-in was performed by targeting and inserting the *ADGB* coding sequence into the AAVS1 site in human chromosome 19 (56). The full-length *ADGB* coding sequence was cloned into the pAAVS1-P-CAG-DEST vector, which is a generous gift from Knut Woltjen (Addgene plasmid # 80490), through Gateway recombination according to the manufacturer's protocol, to generate pAAVS1-P-CAG-ADGB-DEST. Cloned *ADGB* coding sequence is situated downstream of a CMV promoter and Puromycin selection gene, flanked by AAVS-left and -right homology arms. A partnering plasmid pXAT2, which is a generous gift from Knut Woltien (Addgene plasmid # 80494), was co-transfected as the template for AAVS1 sgRNA and Cas9 protein. One day prior to transfection, HEK293T cells, MCF-7 cells, and HeLa cells were seeded on 6-well plates at 5.2 × 10^4^ cells per cm^2^ surface area. Cells were then transfected with 1.2 μg pAAVS1-P-CAG-ADGB-DEST and 1.2 μg pXAT2 using calcium phosphate method of transfection for HEK293T cells (as described above) and ROTIFect for MCF-7 cells and HeLa cells (according to manufacturer's protocol). Upon confluency, cells were subjected to puromycin selection at 2.0 μg/ml for HEK293T and HeLa cells and 1.0 μg/ml for MCF-7 cells. Cells were selected for at least 2 weeks before analysis and continuously maintained under puromycin. *ADGB* knock-in was verified *via* PCR of genomic DNA using primer pair flanking a region within the *ADGB* gene (5′-TGGAGGAGTGTCTTCACCAG-3′), and the AAVS1-right arm (5′-GTCAGAGCAGCT CAGGTTCT-3′). Immunoblotting and immunostaining against ADGB were also performed for verification.

### Immunoprecipitation

Immunoprecipitation was carried out in lysates of HEK293T cells with prior transient overexpression of bait and prey proteins. Cell lysates were prepared using NP-40 lysis buffer (20 mM HEPES/NaOH (pH7.5), 3 mM MgCl_2_, 100 mM NaCl, 1 mM DTT, 1 mM EGTA, 0.5% NP-40, 2 μg mL^−1^ leupeptin, 2 μg mL^−1^ pepstatin, 2 μg mL^−1^ aprotinin, 1 mM PMSF, 1 mM DTT). Cell lysates were subjected to 14,000 RPM centrifugation to recover the supernatant, followed by incubation with 2 μg of primary antibody: anti-GFP (Proteintech), anti-ADGB (Sigma-Aldrich), or anti-rabbit IgG (GE healthcare), at 4 °C for 2 h. The lysates with antibodies were then centrifuged at 14,000 RPM before transferring to 30 μl (bed volume) of Protein G Sepharose 4 Fast Flow (Cytiva) resin and incubated with rotation at 4 °C for 2 h. The resin was then washed 5 times with wash buffer (20 mM HEPES/NaOH (pH7.5), 3 mM MgCl_2_, 150 mM NaCl, 1 mM DTT, 1 mM EGTA, 0.5% NP-40, 2 μg mL^−1^ leupeptin, 2 μg mL^−1^ pepstatin, 2 μg mL^−1^ aprotinin, 1 mM PMSF, 1 mM DTT). To elute the immunoprecipitated proteins, the resin was boiled in equal volume of 2X sample loading buffer (50 mM Tris-HCl, pH 6.8, 2% (w/v) SDS, 10% (v/v) glycerol, 100 mM DTT, 0.004% (w/v) bromophenol blue). The immunocomplexes were analyzed by immunoblotting.

## Data availability

All data are given in the manuscript.

## Supporting information

This article contains supporting information.

## Conflict of interest

The authors declare no conflicts of interest with the content of this article.

## Figures and Tables

**Figure 2 Full Blots:**
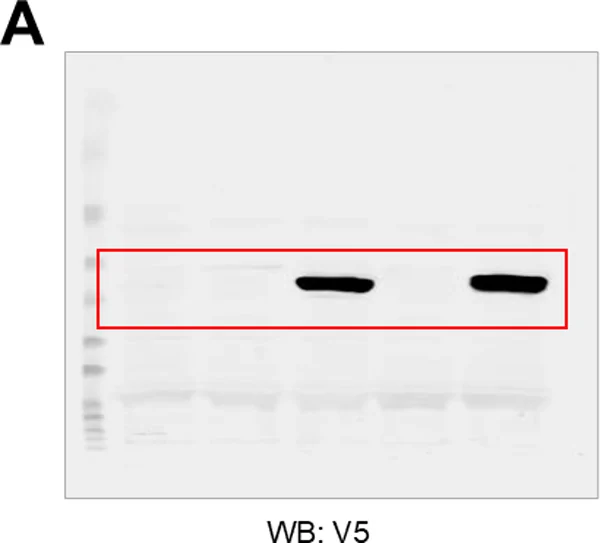


**Figure S2 Full Blots:**
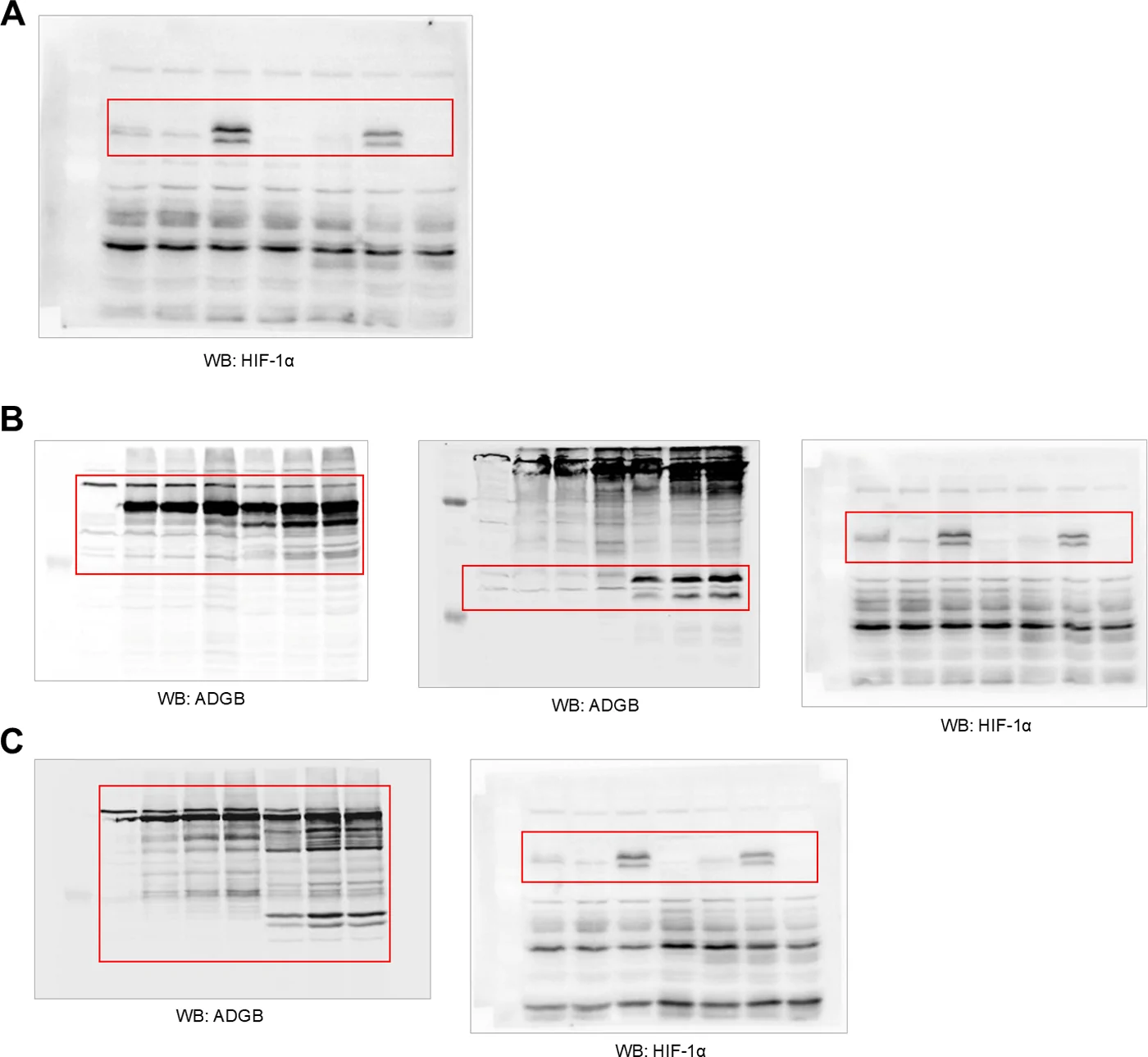


**Figure 3 Full Blots:**
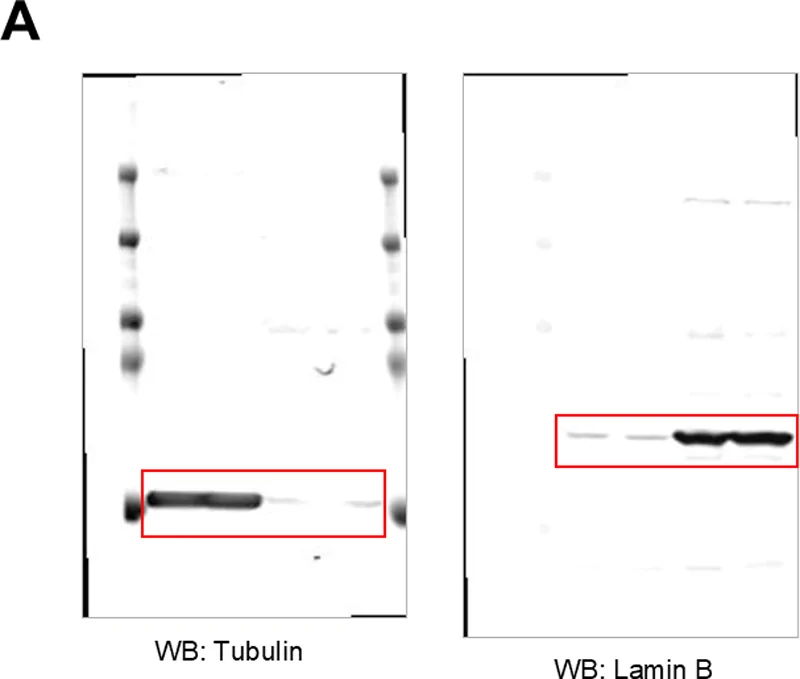


**Figure 4 Full Blots:**
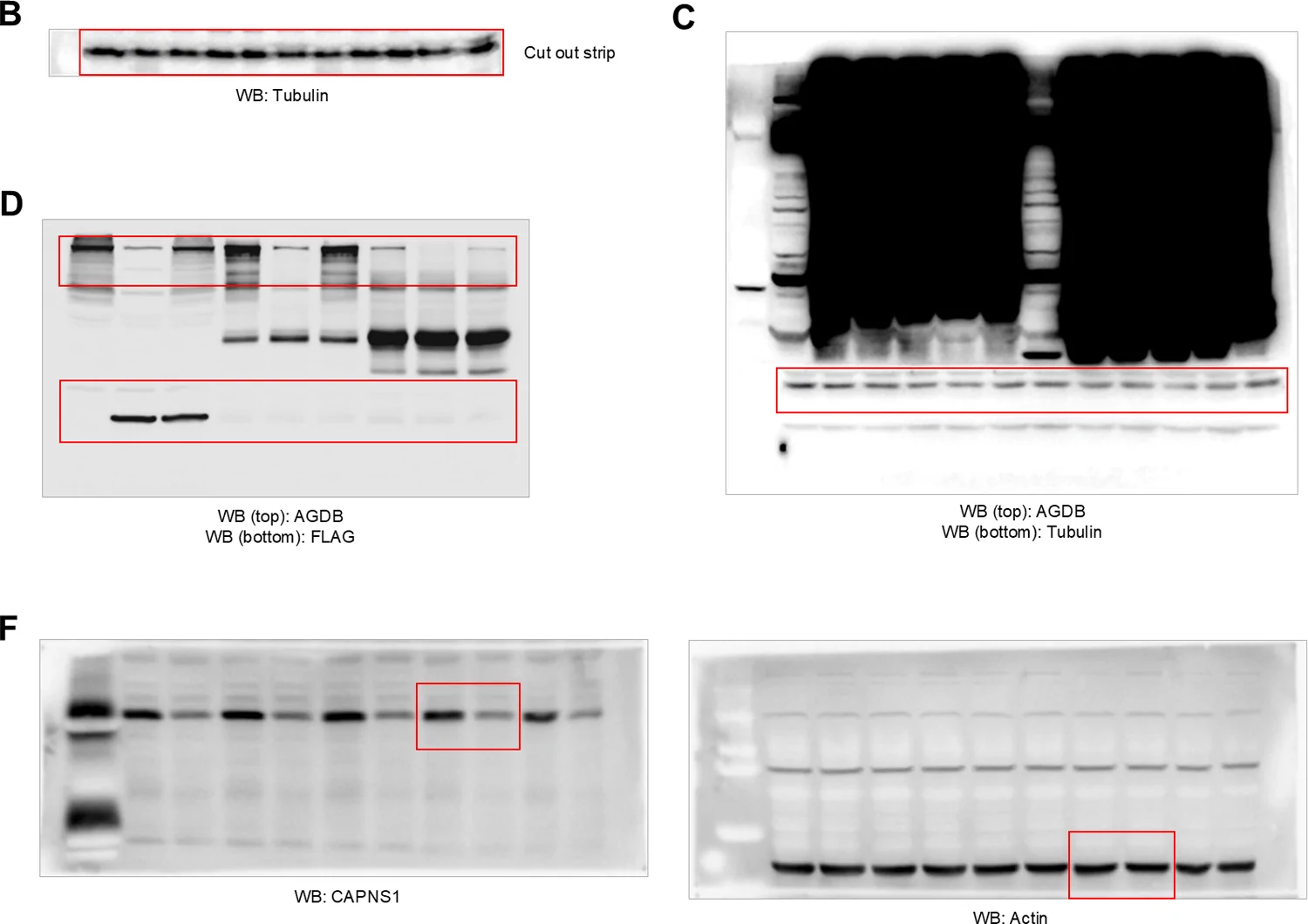


**Figure S4 Full Blots:**
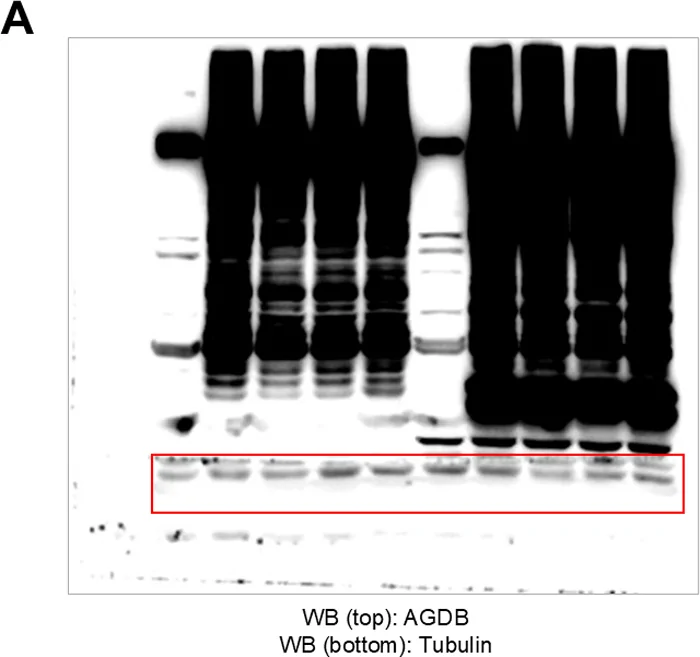


**Figure 6 Full Blots:**
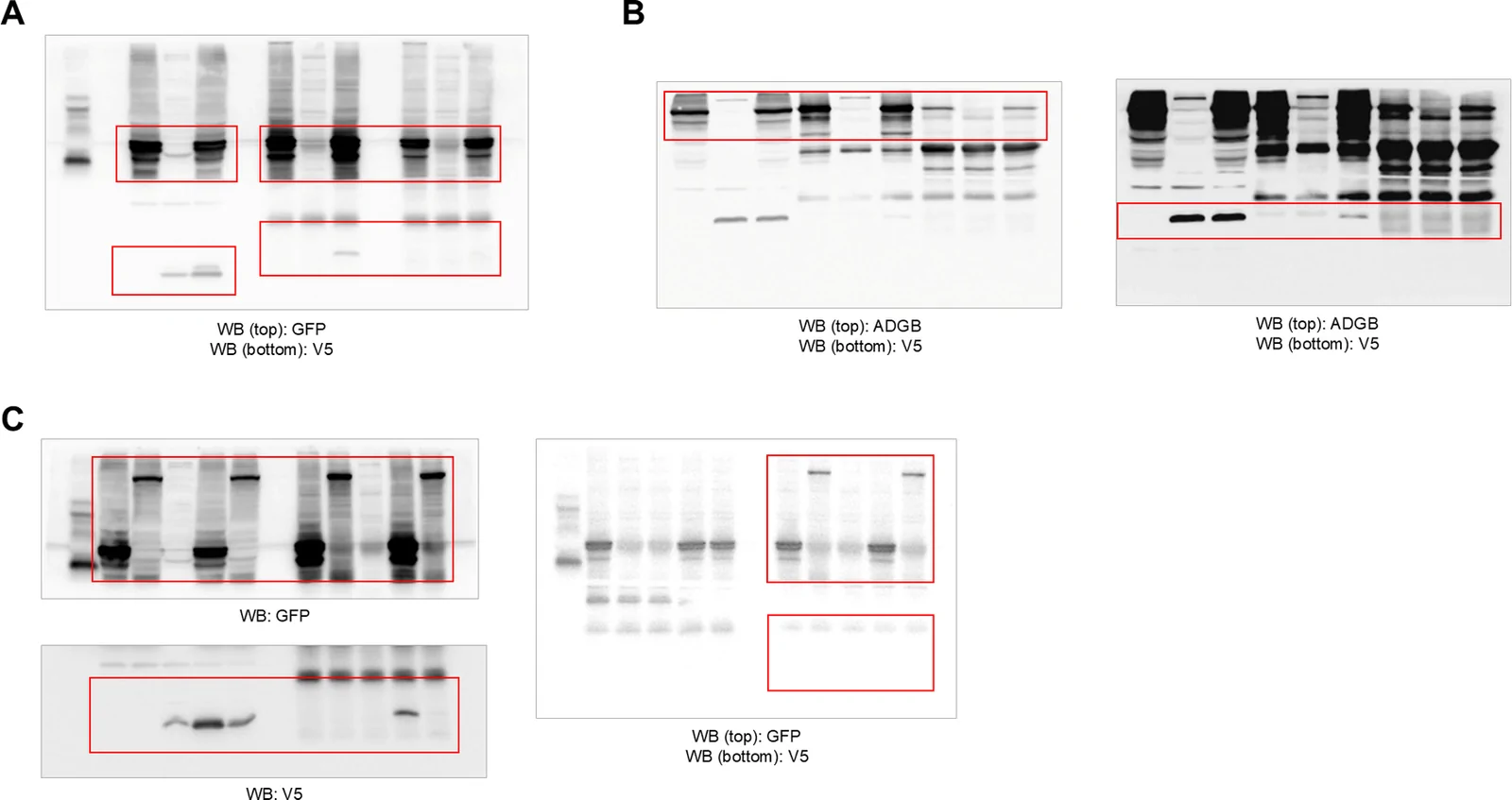


**Figure S9 Full Blots:**