# Supplementary material for: Calcium-dependent proteolysis of the chimeric androglobin reveals altered localization of its isolated globin domain
Source: J Biol Chem. 2026 Sep 18;302(11):113582. doi: 10.1016/j.jbc.2026.113582 (PMC13696810; doi:10.1016/j.jbc.2026.113582)
Supplement: Supplementary file 1 [file mmc1.docx]

**Supporting information**

**Calcium-dependent proteolysis of the chimeric androglobin reveals altered localization of its isolated globin domain**

Teng Wei Koay ^1^, Carina Osterhof ^1^, Angèle Clerc ^1^, David Hoogewijs ^1 §^

^1^ Section of Medicine, Department of Endocrinology, Metabolism and Cardiovascular System (EMC), University of Fribourg, Switzerland

^§^ Corresponding author: David Hoogewijs

E-mail: david.hoogewijs@unifr.ch

**Running title:** Androglobin undergoes calcium-dependent proteolysis

**Supplementary Methods**

**Supplementary Tables**

**Supplementary Figures and legends**

**Supplementary Methods**

**Cloning of recombinant human ADGB gene and the construction of ADGB mutants**

A doubly-tagged recombinant ADGB clone was constructed by the amplification of full-length *ADGB* by PCR with a pair of primers (forward, 5'-AAAAAGCAGGGTCGACCATGGCCTC CAAACAAACCAAAAAG-3', *RE* site underlined; and reverse, 5'-GAAGGCACAG GGTACCTTACAGGTCCTCTTCTGAGATCAGCTTCTGCTCAAAGCTGGGTCTAGATGCCTTTTTCTTTCC-3', *RE* site, underlined, with an additional c-myc sequence included). The amplicon was then digested and ligated into linearized pFLAG-CMV^TM^-6a vector, to generate pFLAG-ADGB-myc. Full-length ADGB used in this study lacks 6 amino acids (res. 26 - 31) at the N-terminal compared to the RefSeq version NM_024694.4. This is highly likely to be arisen from the splicing event as the 6 amino acids are located at the exon-exon splice junction. For consistency, the numbering of ADGB residues will be based on the full-length ADGB sequence as deposited in UCSC Genome Browser. Halo-tag ADGB was generated by amplifying ADGB coding sequence using primer pairs (forward, CAAAAAAGCAGCGATCGCCATGGCCTCC AAACAAACCAAAAAG, *RE* site underlined; reverse, TCAACCACTTGTTTAAACAAAG CTGGGTCTAGATTACTTTTTCTTTCC, *RE* site underlined), and cloned into pFN21A HaloTag® CMV Flexi® Vector (Promega), according to manufacturer’s protocol.

ADGB deletion mutants lacking different domains, the IQ domain (ΔIQ), parts of the uncharacterized 350-residue domains (ΔPro.350res. and ΔDis.350res) and C-terminal domains (ΔPro.C-ter and ΔDis.C-ter), were constructed by ligating amplicons from two parts of the *ADGB* gene with custom designed complementary overhangs. The N-terminal fragments were amplified by PCR using primer pairs (forward, 5'-AAAAAGCAGGGTCGACCATGGCCTCC AAACAAACCAAAAAG-3', *RE* site underlined; and reverse 5' – AATTGCTGCT GGTCTCCTGGCAGCAGAATACTCTTTATCACTTGTGGGCATAC – 3' for ΔIQ, 5' – CTG ATGAGCAGGTCTCAATCCTGCAGAAGAACAATCTGATAGGGACTGC – 3' for ΔPro.350res., 5' – AATAT ATATAGGTCTCATGTGTACACAGAAATCTTCAAAATCTA ACC – 3' for ΔDis.350res., and 5'- ACGGCATGGTGGTCTCGAGCAATCTTAGTTTCTTC ATCTTG -3' for ΔPro.C-ter, *RE* site underlined). Similarly, the C-terminal fragments were amplified by PCR using primer pairs (forward, 5'- CAGATTGTTCGGTCTCAATACAGACC TCTCATATGGTCGTATATGC-3' for ΔProtease, 5' – GCTATCAAGATGGGTCTCCCAT AATTGCTTTTGCAGATTATACTGTGAC – 3' for ΔGlobin , 5' – TTAGATTGCTGGTCTC AGCCAGAATACCAGACACAAAAGAAAATATCAGTG – 3' for ΔIQ, 5' – ATGAACAAA CGGTCTCTGGATTGGGTGATGCTCATCAGAGTGATG – 3' for ΔPro.350res., 5' – CAGT AGGACAGGTCTCACACATCTGCAGCATGGTGTC – 3' for ΔDis.350res., and 5'- TATGG TGAAAGGTCTCATGCTTTAATTAACTTAGGAAGCCCAGACTCC -3' for ΔPro.C-ter, *RE* site underlined; and reverse 5'-GAAGGCACAGGGTACCTTACAGGTCCTCTTCTGA GATCAGCTTCTGCTCAAAGCTGGGTCTAGATGCCTTTTTCTTTCC-3', *RE* site underlined, with an additional c-myc sequence included). For ΔDis.C-ter, single amplicon was amplified by PCR using the primer pairs (5'-AAAAAGCAGGGTCGACCATGGCC TCCAAACAAACCAAAAAG-3' *RE* site underlined; and reverse, 5'- AAGATTTTTGGGTA CCTTACAGGTCCTCTTCTGAGATCAGCTTCTGCTCGGCGGCCTCCTCGTGTCTTTCACCATAAGC -3', *RE* site underlined, with an additional c-myc sequence included). These amplicons were then digested with their respective restriction enzymes as designed on the primers, and ligated (2- or 3-way ligation) into linearized pFLAG-CMV^TM^-6a vector digested with *Xho*I and *RE*, to generate pFLAG-ADGBΔProtease-myc (res. 57 – 409 removed), pFLAG-ADGBΔGlobin-myc (res. 763 – 985 removed), pFLAG-ADGBΔIQ-myc (res. 905 – 927 removed), pFLAG-ADGBΔPro.350res-myc (res. 410 – 588 removed), pFLAG-ADGBΔDis.350res-myc (res. 641 – 762 removed), pFLAG-ADGBΔPro.C-ter-myc (res. 988 – 1295 removed), and pFLAG-ADGBΔDis.C-ter-myc (res. 1296 – 1667 removed). All deletion mutants contain a FLAG-tag and a c-myc-tag at the N-terminus and the C-terminus, respectively. Generation of ADGB-ΔIQ and ADGB-Δcalpain constructs were described before (Keppner et al. 2022)

ADGB truncated mutants consisting of only the N-terminal domain (res. 1 – 57) and the C-terminal domain (res. 969 – 1667) were cloned. These truncates were designed to carry a FLAG-GFP tag at the N-terminal and GFP tag at the C-terminal with glycine-serine linkers (GGSGGGGSGG) between the tags and the ADGB domains. The N-terminal GFP tag was amplified by PCR using a primer pair (forward, 5'- GCTAGTTAAGTCGACCATGGTGAGC AAGGGCGAGGAG-3', *RE* site underlined; reverse, 5'- ATATAGGTCTCA**TCCTCCACT ACCACCACCACCACTACCTCC**CTTGTACAGCTCGTCCATGCCGAG, *RE* site underlined, glycine-serine linker in bold). Similarly, the C-terminal GFP tag was amplified using a primer pair (forward, 5'- GACTCGGTCTCG**GGAGGTAGTGGTGGTGGTGGTA GTGGAGGA**ATGGTGAGCAAGGGCGAGGAG -3', *RE* site underlined, glycine-serine linker in bold; reversed, 5'- TGTACAAACTGGTACCTTACTTGTACAGCTCGTCCATGCC GAG -3', *RE* site underlined). Truncates of ADGB domains with complementary overhangs to the above N- and C-terminal tags were amplified using primer pairs, for N-terminal domain (forward, 5'- CACAAGGGTCTCCAGGAATGGCCTCCAAACAAACC-3'; reverse, 5'- GCA TCCGGTCTCTCTCCATTTATGTCAGCTTCACTCC -3', *RE* site underlined), and the C-terminal domain (forward, 5'- GACTAAGGTCTCAAGGAAAGTGCAAGTCTTTGGAATC -3'; reverse, 5'- GAGATCGGTCTCTCTCCAAAGCTGGGTCTAGATGCC -3', *RE* site underlined). These amplicons were then digested with their respective restriction enzymes as designed on the primers, and ligated (4-way ligation) into linearized pFLAG-CMV^TM^-6a vector, to generate pFLAG-GFP-Nter-GFP (res. 1 – 57, without res. 26 – 31) and pFLAG-GFP-Cter-GFP (res. 969 – 1667).

Single GFP-tagged full length ADGB, globin domain (ADGB_GD), and globin domain with C-terminal segment (ADGB_GD-Cter) were cloned by using the above mentioned N-terminus GFP-tagged to ligate with amplicons amplified by PCR using the primer pairs for full length ADGB (forward, 5'- CACAAGGGTCTCCAGGAATGGCCTCCAAACAAACC-3, *RE* site underlined; reverse, reverse, 5'-GAAGGCACAGGGTACCTTACAGGTCCTCTTCTGAGA TCAGCTTCTGCTCAAAGCTGGGTCTAGATGCCTTTTTCTTTCC-3', *RE* site underlined), ADGB_GD (forward, CAGTAGGGTCTCCAGGACACATCTGCAGCATGGTGTC, *RE* site underlined; reverse, ACCACCGGTACCTTAGCTTTTAAACATTAGTCTTAAGAGAGAA ACTGC, *RE* site underlined), and ADGB_GD-Cter (forward, CAGTAGGGTCTCCAGGAC ACATCTGCAGCATGGTGTC, *RE* site underlined; reverse, 5'-GAAGGCACAGGGT ACCTTACAGGTCCTCTTCTGAGATCAGCTTCTGCTCAAAGCTGGGTCTAGATGCCTTTTTCTTTCC-3', *RE* site underlined). Digested amplicons were ligated into linearized pFLAG-CMV^TM^-6a vector to generate pFLAG-GFP-ADGB, pFLAG-GFP-ADGB_GD, and pFLAG-GFP-ADGB_GD-Cter. With the same construction to pFLAG-GFP-ADGB_GD, GFP-tagged ADGB globin domains with single mutation on the proximal histidine (H824G) or distal glutamine (Q792G), or both (H824G/Q792G) were cloned by amplifying and ligating the globin gene using primers designed to carry the mutated codon sequence.

FLAG-tagged ADGB N-terminal segment consisting of the res. 1 to 987 of the protein was cloned by ligating linearized pFLAG-CMV^TM^-6a vector with digested amplicon amplified using primer pairs (forward, 5'- GCTAGTTAAGTCGACCATGGTGAGCAAGGGCGAGG AG-3', *RE* site underlined; reverse, ACCACCGGTACCTTAGCTTTTAAACATTAGTCTTA AGAGAGAAACTGC, *RE* site underlined), to generate pFLAG-Nter-ADGB_GD.

For protein purification, full-length ADGB with GST-tag at the N-terminus was constructed, with a glycine-serine linker (GGSGGGGSGG) between the GST-tag and the ADGB. The N-terminal GST-tag was amplified by PCR, with pDEST^TM^20 vector (Invitrogen) as a template, using a primer pair (forward, 5’- CGTACGGTCGACCATGGCCCCTATACTA GGTTA -3’, *RE* site underlined; reverse, 5’- ATATAGGTCTCA**TCCTCCACTACCACCACCACCAC TACCTCC**TTTTGGAGGATGGTCGCCAC -3’, *RE* site underlined, glycine-serine linker in bold). Full-length ADGB was amplified with a primer pair (forward, 5’- AATAAGGGTCTC CAGGAATGGCCTCCAAACAAACCAAAAAG -3’, *RE* site underlined, reverse, 5’- ACCA CTGGTACCTTAAAAGCTGGGTCTAGATGCCTTTTTCTTTCC -3’, *RE* site underlined). These amplicons were then digested with their respective restriction enzymes as designed on the primers, and ligated (3-way ligation) into linearized pFLAG-CMV^TM^-6a vector to generate pFLAG-GST-ADGB.

For the study of ADGB globin and IQ domain, ADGB mutant with R918Q/R923Q were synthesized in pENTR^TM^4 vector, and the gene was sub-cloned into pcDNA3.1-EGFP Destination vector, according to manufacturer’s protocol, to generate pcDNA3.1-EGFP-ADGB(IQ.RR) plasmid.

**Cloning of hSpec cleavage site**

The amplicon for hSpec fragment was generated from synthesized complementary oligo strands complexed in T4 DNA ligase buffer (ThermoScientific) (40 mM Tris-HCl, 10 mM MgCl_2_, 10 mM DTT, 500 μM ATP) with T4 poly nucleotide kinase (ThermoScientific) at 37°C for 1 hour, followed by heating to 95°C for 5 minutes and slow cooling at the rate of -5°C min^-1^ to 10°C, to produce oligo duplex (with *EcoR*I overhangs at both ends). The oligo duplex is then cloned into pM3-Gal4BD-VP16AD vector linearized with *EcoR*I digestion, generating pM3-Gal4-hSpec-VP16 (insert: *GSG*QQEVYGMMPRD*GSG*, hSpec underlined, glycine-serine linker *italized*).

**Antibodies**

The antibodies used in this study include anti-ADGB (1:500) (Sigma-Aldrich, HPA036340-100UL), anti-GFP (1:1000) (Proteintech, 50430-2-AP-150UL), anti-FLAG (1:1000) (Proteintech, 20543-1-AP (rabbit)/ 66008-3-IG-150UL (mouse)), anti-GST (1:500) (OriGene, TA150033), anti-tubulin (1:1000) (Santa Cruz, sc-23950), anti-laminB (1:1000) (abcam, ab16048), anti-HIF1α (1:1000) (BD Transduction Laboratories™, 610958), and anti-V5 (1:1000) (Proteintech, 14440-1-AP-150UL).

**Table S1:** List of centrosomal and/or ciliary proteins co-immunoprecipitated with the isolated globin domain

| Protein | Enrichment in Glob-GFP (log2) | GO centrosome | GO_ciliary basal body | HPA location centrosome | HPA location basal body | Comments |
| --- | --- | --- | --- | --- | --- | --- |
| CCT8 | 6.08 | y | n | n | n | localisation to sperm connecting piece |
| TCP1 | 4.68 | y | n | - | - | no data |
| EMD | 4.48 | y | n | n | n |  |
| RUVBL1 | 4.27 | y | y | n | y |  |
| CALM1 | 4.18 | y | n | n | n |  |
| BIRC6 | 3.98 | y | n | n | n |  |
| KIF11 | 3.90 | n | y | n | y | localisation to sperm midpiece |
| ESPL1 | 3.76 | y | n | n | n |  |
| PCNA | 3.69 | y | n | n | n |  |
| ALMS1 | 3.54 | y | y | y | y |  |
| BCAS2 | 3.39 | y | n | y | n |  |
| IQSEC1 | 3.20 | y | n | y | n |  |
| SLC16A1 | 3.14 | y | n | n | n |  |
| STIL | 3.13 | y | y | y | y |  |
| SLC1A5 | 3.10 | y | y | y | y | localisation to centriolar satellite, uncertain |
| RBM39 | 3.08 | y | n | y | n | localisation to centriolar satellite, uncertain |
| RPAP3 | 3.01 | n | y | n | y | localisation to sperm acrosome |
| SPAG5 | 2.92 | y | y | n | n |  |
| ERC1 | 2.90 | y | y | y | n |  |
| MMS19 | 2.84 | y | n | n | n |  |
| CEP85 | 2.61 | y | n | y | n |  |
| DNM2 | 2.52 | y | n | n | n |  |
| HAUS5 | 2.52 | y | n | - | - | no data |
| USP9X | 2.33 | y | n | n | n |  |
| NCAPD2 | 2.29 | y | y | y | y | localisation uncertain |
| OBSL1 | 2.28 | y | n | y | n |  |
| PIK3R4 | 2.26 | n | y | n | y | localisation uncertain |
| TUBGCP2 | 2.13 | y | y | y | y |  |
| KRT18 | 2.11 | y | n | n | n |  |
| PSMB5 | 2.04 | y | n | y | n | localisation to sperm midpiece |
| PSMA1 | 2.00 | y | n | y | n | localisation uncertain |
| WDR62 | 1.95 | y | n | y | n | localisation to centriolar satellite |
| CDK5RAP2 | 1.94 | y | y | y | y | localisation to sperm midpiece |
| PKN2 | 1.86 | y | n | y | n |  |
| CCT5 | 1.80 | y | n | n | n | localisation to sperm midpiece |
| CCT4 | 1.72 | y | n | n | n |  |
| HAUS6 | 1.65 | y | n | y |  | localisation to centriolar satellite & sperm midpiece |
| ACLY | 1.60 | n | y | n | y | localisation to flagellar endpiece, uncertain |
| DCTN1 | 1.56 | y | y | n | y |  |
| WDR11 | 1.48 | n | y | y | n | localisation to flagellar centriole & principal piece |
| DYNC1H1 | 1.48 | y | n | n | n | localisation to flagellar centriole & midpiece |
| DYNC1I2 | 1.42 | y | n | y | n | localisation to flagellar centriole & midpiece |
| RRM1 | 1.39 | y | y | y | y | localisation to centriolar satellite, uncertain |
| CTDP1 | 1.38 | y | n | n | n |  |
| YTHDF2 | 1.36 | y | n | n | n | cell-line dependent localisation to centriolar satellite |
| LUZP1 | 1.26 | y | y | y | n |  |
| ATXN10 | 1.23 | n | y | n | n |  |
| FLII | 1.23 | y | n | y | n | localisation to centriolar satellite |
| SKP1 | 1.18 | y | n | y | y |  |
| NEK9 | 1.17 | y | n | - | - | no data |
| TUBGCP6 | 1.04 | y | n | - | - | no data |
| KIF7 | 1.02 | n | y | n | n |  |

**Table S2. qPCR primers**

| Target | Primer fwd | Primer rev |
| --- | --- | --- |
| ACTIN | CTG GAA CGG TGA AGG TGA CA | AAG GGA CTT CCT GTA ACA ATG |
| CAPN1 | AGA GTG GAA CAA CGT GGA CC | AAG GTG GCT GGG TAG TTT CG |
| CAPN2 | AAGTAACGGAAGCCTACAGAAAC | ATCTTCATGCCGTCTGGTCAG |
| CAPNS1 | CGT GAT GGA TAG CGA CAC CA | TCA TTC AGG TGG AAC CCT GC |

**Supplementary figures:**

**Figure S1. ADGB exhibits calcium-mediated proteolysis in a wide variety of experimental settings.** In addition to the data presented in Figure 1, GFP-ADGB (A), FLAG-GST-ADGB (B), and Halo-ADGB (C) were transiently expressed in HEK293T cells with and without the treatment of 50 mM CaCl_2_/ 25 μM calcium ionophore, and 100 μg of total cell lysate were analyzed by immunoblotting with an antibody against ADGB. Arrows indicate the full length ADGB with their respective protein-tag as designed in the plasmid. Open and closed triangles indicate cleaved ADGB products detected following calcium treatment. Asterisk indicates a small cleaved ADGB product that is only noticeable with the FLAG-ADGB-myc, which has smaller tags at both N- and C-termini. (D, E) Calcium-mediated ADGB cleavage is also observed in ADGB knock-in cellular models with constitutive ADGB expression. Full length ADGB is indicated by an arrow, and cleavage products with a corresponding migration pattern are indicated by open triangles in HeLa (D), and MCF-7 (E).

**Figure S2. Calcium-mediated ADGB proteolysis remains detectable following deletion of the ADGB globin domain, calpain-like domain, or IQ motif and under different oxygen conditions.** (A) FLAG-ADGB-myc was transiently expressed in HEK293T cells under normoxia (atmospheric O_2_ levels), hypoxia (0.2% O_2_), and hypoxia followed by reoxygenation (ROS stress). For oxygen deprivation, transfected cells were incubated under hypoxia for 24 hours and reoxygenation was carried out for 1 hour at normoxia prior to cell lysis. For samples with calcium treatment, cells were treated with 50 mM CaCl_2_/ 25 μM calcium ionophore for 1 hour prior to cell lysis. 100 µg of cell lysate were analyzed by immunoblotting with antibodies against ADGB and HIF-1α. The presence of HIF-1α in hypoxia and not in the oxygenated samples was used as a marker for oxygen deprivation. Full-length ADGB displayed a comparable calcium-responsive cleavage pattern under normoxia, hypoxia and reoxygenation. The arrow indicates full length ADGB, whereas the closed triangle, open triangle and asterisk indicate cleaved ADGB products. (B) Total deletion of the globin domain in ADGB did not prevent detection of calcium-responsive ADGB cleavage products The larger cleaved products indicated by closed triangle ([’] for mutant) displayed a downward shift in apparent molecular weight corresponding to the globin-domain deletion, whereas the smaller cleavage products, indicated by open triangle and asterisk, showed comparable migration to those detected for WT ADGB. (C-D) FLAG-ADGB-myc was transiently expressed in HEK293T cells in normoxia (atmospheric O_2_ level), hypoxia (0.2% O_2_), and hypoxia followed by reoxygenation (ROS stress), with subsequent analysis as described in (A). Calcium-responsive cleavage products remained detectable under the oxygen conditions tested in both the protease-domain deletion mutant (C) and the IQ-motif deletion mutant (D).

**Figure S3.** **Recombinant ADGB does not exhibit instantaneous cleavage in the presence of calcium.** *In vitro* autolysis assay of purified FLAG-GST-ADGB (~1.2 μg) with and without 10 mM CaCl_2_, incubated at 37°C for 3.5 or 22 hours. Full-length ADGB remained stable, and proteolytic products were not detected under any of the conditions tested.

**Figure S4. Effects of PD150606 and MG-132 on calcium-mediated ADGB cleavage.** (A) FLAG-ADGB-myc was transiently expressed in HEK293T cells with and without 50 mM CaCl_2_/ 25 μM calcium ionophore treatment, along with increasing dosage of CAPN1- and CAPN2-specific inhibitor, PD150606 (Chukai et al., 2021; Low et al., 2014). No appreciable alteration in the calcium-responsive ADGB cleavage pattern was observed across the concentrations of PD150606 tested. Full length ADGB is indicated by arrow, whereas cleaved products are indicated by closed triangle, open triangle, and asterisk. Loading control is shown with an immunoblot using anti-tubulin antibody. (B) FLAG-ADGB-myc was transiently expressed in HEK293T cells and treated with 10 μM MG-132 (inhibitor of proteasomes, serine proteases, calpains, and other proteases) for 23 hours prior to 1-hour treatment with 50 mM CaCl_2_/ 25 μM calcium ionophore. Following MG-132 treatment, the calcium-responsive ADGB cleavage products displayed a qualitatively altered band pattern, with less prominent cleavage-product bands in the representative immunoblot. (C) The efficiency of PD150606 in inhibiting CAPN1/2 is verified using the mammalian-1-hybrid system with Gal4-hSpec-VP16 and pGRE5xE1b in a luciferase-based read-out. Active proteolytic activity of calpains cleaves human spectrin (hSpec) and separates Gal4 and VP16, leading to lower transcriptional activation of pGRE5X. Results are displayed as ratios of firefly to *Renilla* luciferase activities in relative light units (RLU) and normalized to the pGRE5xE1b promoter alone without Gal4-hSpec-VP16. Increasing dosage of PD150606 results in increasing pGRE5xE1b promoter-driven luciferase activity (N=3).

**Figure S5.** **RT-qPCR analysis confirms downregulation of CAPN1, CAPN2, and CAPNS1 following esiRNA treatment in HEK293T cells.**

**A**

**B**

**C**

**Figure S6. Comparison of predicted calpain cleavage sites with experimentally inferred regions of ADGB processing.** (A) DeepCalpain-predicted cleavage sites show a cluster within amino acids 550–650 of ADGB, overlapping with a region implicated by the deletion-mapping experiments. (B) Schematic representation of the approximately 60-kDa and 135-kDa fragments observed following calcium-mediated ADGB cleavage. (C) Schematic inference of candidate calcium-responsive cleavage region(s) based on the apparent molecular-weight shifts of the approximately 60-kDa and 135-kDa fragments following domain deletion.

**Figure S7. Schematic representation of ADGB mutants used in this study.**

**Figure S8. The truncated ADGB N-terminal region displays calcium-dependent proteolytic processing.** The ADGB N-terminus segment flanked with FLAG-GFP at the N-terminus and GFP at the C-terminus was overexpressed in HEK293T cells with and without treatment of 50 mM CaCl_2_/ 25 μM calcium ionophore. Immunoblotting analysis against GFP and FLAG shows calcium dependent proteolysis in this segment. Dual-mode imaging of GFP and FLAG was used to differentiate the N-terminal fragments (with FLAG tag) and the C-terminal fragment (without FLAG tag). The full-length protein and its cleaved product are indicated by arrow and closed triangle, respectively.

**Figure S9.** **Interaction of the ADGB globin domain with calmodulin depends on the position of the GFP tag.** Co-immunoprecipitation of FLAG-GFP-ADGB_GD or FLAG-ADGB_GD-GFP (with GFP at the C-terminal of the globin domain instead of the N-terminal) and V5-CALM in HEK293T cells. Recombinant proteins were transiently expressed and cell lysates were then incubated with anti-rabbit IgG (negative control) or anti-GFP antibody. The immunoprecipitates were immunoblotted against GFP and V5 with 2% cell lysates as input. The presence of C-terminal GFP interfere with the binding of V5-CALM onto the ADGB_GD, whereas the absence of GFP protein at the C-terminal of ADGB globin domain allows the interaction with V5-CALM, as indicated in the immunoblot against V5.

**Figure S10. Preferential centrosomal localization of the isolated ADGB globin domain remains detectable following mutation of heme-binding residues.** FLAG-GFP-ADGB_GD-WT or FLAG-GFP-ADGB_GD-F8(Gly) or GFP-ADGB_GD-E8(Gly) or FLAG-GFP-ADGB_GD-F8/E7(Gly) were transiently expressed in HEK293T cells for 24 hours prior to 3.7% PFA/PBS fixation and mount with DAPI staining. GFP signals displaying the characteristic centrosomal/pericentrosomal localization pattern were observed for WT and mutant constructs. Representative centrosomal signals are indicated by white triangles.
